# A Mediterranean Diet‐Based Food Mix Ameliorates Diabetes‐ and Obesity‐Associated Liver Alterations Through Mitochondrial and Metabolic Reprogramming

**DOI:** 10.1002/mnfr.70210

**Published:** 2025-09-01

**Authors:** Giovanna Mercurio, Antonia Giacco, Antonio Dario Troise, Moira Ledbetter, Sabrina De Pascale, Nicla Scopigno, Michela Vigliotti, Matteo Mazzola, Giuseppe Riccio, Andrea Scaloni, Maria Moreno, Alberto Fiore, Federica Cioffi, Elena Silvestri

**Affiliations:** ^1^ Department of Science and Technology University of Sannio Benevento Italy; ^2^ Proteomics Metabolomics & Mass Spectrometry Laboratory Institute for the Animal Production System in the Mediterranean Environment National Research Council Portici Italy; ^3^ Division of Engineering and Food Science School of Applied Sciences University of Abertay Dundee Scotland; ^4^ Cardiothoracic and Vascular Department Azienda Ospedaliero‐Universitaria Pisana Pisa Italy; ^5^ I.SA.MA S.r.l. Sant'Egidio del Monte Albino, Salerno (SA) Italy

**Keywords:** autophagy, mitochondrial biogenesis, mitochondrial dynamics, mitophagy, OXPHOS

## Abstract

Metabolic syndrome (MetS)‐related diseases, such as type 2 diabetes (T2D) and obesity, are among the leading causes of liver damage, and their prevalence poses an increasing clinical challenge. The Mediterranean diet (MD) has shown promising effects in managing MetS, reducing mortality and morbidity. However, the precise biochemical and molecular mechanisms underlying the MD efficacy remain unclear. This study evaluated the effects of a lab‐designed balanced food mix, simulating the 1960s MD, on hepatic metabolic dysfunction in obese and diabetic db/db mice.

The MD cocktail, naturally rich in polyphenols, fructose, and monounsaturated fatty acids, prevented hyperlipidemia while not reversing diabetes and obesity. Gene expression, protein representation, and metabolomic analyses of liver tissues from MD‐fed db/db mice revealed reduced oxidative damage, preserved mitochondrial quality control, enhanced autophagy markers, and reduced fibrosis markers. The MD cocktail also enhanced liver mitochondrial mass and stimulated the OXPHOS system. It also preserved the hepatic pool of acylated carnitine derivatives and chenodeoxycholic acid, suggesting protective effects on mitochondrial β‐oxidation and bile acid biosynthesis, with an overall improvement of metabolite profiles.

The experimental MD cocktail exerted significant hepatoprotective effects, mitigating several diabetes‐ and obesity‐induced hepatic disturbances and beneficially affecting metabolic fluxes and tissue texture.

## Graphical abstract

Metabolic syndrome (MetS)‐related conditions are key factors in liver damage. A Mediterranean lifestyle shows promise in managing MetS, though its mechanisms remain unclear. This study tested a lab‐designed food mix mimicking the 1960s Mediterranean Diet (MD) on obese and diabetic mice; it prevented hyperlipidemia and reduced oxidative liver damage, improved mitochondrial function, metabolomic profiles and autophagy, and decreasing fibrosis. Overall, the MD cocktail showed hepatoprotective effects.

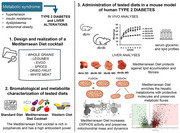

**Abbreviations**
ABCG5: ATP binding cassette subfamily G member 5
ABTS: 2,2′‐azinobis(3‐ethylbenzothiazoline‐6‐sulfonic acid)
ACC1: acetyl‐CoA carboxylase
AMBRA: activating molecule in Beclin1‐regulated autophagy
AMPK: 5' AMP‐activated protein kinase
BN‐PAGE: blue‐native polyacrylamide gel electrophoresis
CAT: catalase
CD36: cluster of differentiation 36
CHREBP1: carbohydrate response element binding protein 1
COLIV: collagen type IV
COX‐II: subunit II of cytochrome c oxidase
CYP7A1: cholesterol 7 alpha‐hydroxylase
CYTB: cytochrome B
DGAT: diacylglycerol o‐acyltransferase
DPPH: 2,2 diphenyl‐1‐1picrylhydrazyl
DRP1: dynamin‐related protein 1
FAS1: fatty acid synthase 1
FRAP: ferric reducing power
G6P: glucose‐6‐phosphatase
GLY: fasting glucose
GOT: glutamate oxaloacetate transaminase
GPX4: glutathione peroxidase 4
HBA1C: glycated hemoglobin
HIF1α: hypoxia‐inducible transcription factor
HMGCR: 3‐hydroxy‐3‐methylglutaryl‐CoA reductase
IL‐6: interleukin 6
LC3B‐II: proteins 1A/1B light chain 3B
LDLR: low‐density lipoprotein receptor
LPL: lipoprotein lipase
MD: Mediterranean diet
MetS: metabolic syndrome
Mt‐DNA: mitochondrial DNA
MT‐MMP1: membrane‐type matrix metalloproteinase 1
mTOR: mammalian target of rapamycin
NAD: nicotinammide adenina dinucleotide
NADH: reduced nicotinamide adenine dinucleotide
NAFLD: nonalcoholic fatty liver disease
OXPHOS: mitochondrial oxidative phosphorylation system
PCK1: phosphoenolpyruvate carboxykinase 1
PGC1α: peroxisome proliferator‐activated receptor gamma coactivator 1‐alpha
PINK1: PTEN‐induced kinase 1
PPAR: peroxisome proliferator‐activated receptor
PRDX3: peroxiredoxin 3
PS: phosphatidylserine
RAGE: receptor for advanced glycation end‐product
ROSreactive oxygen species: reactive oxygen species
SD: standard diet
SFA: saturated fatty acid
SOD: superoxide dismutase
SREBP: transcription factor sterol regulatory element binding protein
T2D: type two diabetes
TFAM: mitochondria transcription factor A
TG: triglyceride
TIMP2: tissue inhibitor of metalloproteinase‐2
TMAO: trimethylamine‐N‐oxide
TPC: total phenolic content
VDAC: voltage‐dependent anion channel
VEGF: vascular endothelial growth factor
WD: Western diet

## 1 Introduction

The prevalence of metabolic syndrome (MetS) and its associated comorbidities has surged in recent years, yet effective long‐term treatments remain elusive. Despite ongoing research, many pharmacological interventions fail due to adverse side effects, leaving lifestyle modifications as the most effective approach to reduce complications such as type 2 diabetes (T2D) [1] and liver conditions [2]. In this scenario, the adherence to the Mediterranean diet (MD) plays a key role. Since Ancel Keys' studies [3], the MD has been linked to human longevity and is widely recommended for a healthy individual condition. However, its role in pathological conditions remains debated. The MD has shown promising results in reducing mortality and improving clinical outcomes in MetS patients, specifically in nonalcoholic fatty liver disease (NAFLD) [4, 5, 6, 7]. However, the specific cellular and intra‐organ mechanisms underlying the systemic effects of the MD remain unclear, and many questions about its efficacy and activity remain unanswered. Indeed, despite the demonstrated effects of various bioactive compounds within MD foods—such as activation of antioxidant enzymes [8, 9], regulation of AMP‐activated protein kinase (AMPK), mammalian target of rapamycin (mTOR), and peroxisome proliferator‐activated receptor‐γ coactivator 1α (PGC‐1α) pathways [10, 11], and enhanced mitochondrial respiratory activity [12, 13]—the majority of studies have utilized in vitro models in combination with plant extracts and purified substances. In a similar fashion, the Western diet (WD) provides high caloric density, saturated lipids, and oxidized compounds that can impact cellular mechanisms and alter the gut microbiota with undesired consequences at organ levels [14]. The alteration of lipid and glucose metabolism, along with oxidative stress—biochemical and molecular hallmarks of T2D and obesity—has been shown to have a profound impact on mitochondrial function, with pathological consequences such as fibrosis [15, 16, 17]. This study tackles MD principles in the nutritional management of diabetes and obesity, deciphering the putative organ‐protective effects of a lab‐designed, balanced food mix in the form of an experimental cocktail for rodents, simulating the MD of the 1960s [18, 19]. This MD cocktail was compared to both a standard diet (SD) and a WD for its nutrient composition, phytochemical content, antioxidant properties, and overall metabolic effects. The different diets were comparatively assessed in an animal model of spontaneous diabetes and obesity (db/db mouse) [20], with a focus on the liver and mitochondria to get further insights into the molecular mechanisms behind the protective effects of MD toward the above‐reported metabolic diseases. Thus, control and diabetic/obese animals were initially monitored for body weight, food consumption, blood glucose, and lipid parameters. Liver‐specific effects of MD, SD, and WD were further evaluated through the analysis of expression of markers related to tissue damage, oxidative stress, and mitochondrial quality control. Additional assessments included liver gross morphology, triglyceride content, gene expression analysis of markers of lipid metabolism, and liver metabolomics to outline the interconnection between diet and liver health.

## 2 Materials and Methods

### 2.1 Ethics Statement

The mouse protocols were approved by the Ethics Committee of Biogem (IRGS, Ariano Irpino, 83031 Avellino, Italy). All experiments were performed in accordance with the guidelines approved by the Italian Ministry of Health (project authorization number 711/2020‐PR). As suggested by the animal welfare protocol, all efforts were made to minimize animal suffering and to use only the number of animals necessary to produce reliable scientific data.

### 2.2 Diets and Animal Model Design

Three diets were used: a control item and two test diets, namely a standard diet (SD; 62% carbohydrates, 27% proteins, 11% fat, 16.3 kJ/g), an original lab‐designed Mediterranean diet cocktail (MD; 46% carbohydrates, 17% proteins, 37% fat, 16.7 kJ/g), and a Western diet (WD; 43% carbohydrates, 15% proteins, 42% fat, 18.8 kJ/g). All diets were purchased from Mucedola S.r.l. (Milano, Italy). The MD cocktail was produced using food raw materials (mainly from Campania, Italy) [whole grains (29%, dry weight) (wheat, spelt, barley, rye, oats, millet), legumes (10%) (beans, chickpeas, lentils, and peas), cod (8%), freeze‐dried chicken (8%), dried tomatoes (7%), extra virgin olive oil (EVOO) (11%), dried fruits (24%) (apple, walnuts, hazelnuts), rosemary, and onion (3%)], mixed together and pelleted for animal diet suitability by Mucedola S.r.l., following an original recipe developed according to scientific publications on MD composition [18, 19]. The pelleting process by cold drawing did not involve heating. The drying step was carried out using hot air at a maximum temperature of 32°C until a final moisture content of 12% was reached. No additional processing or thermal treatment was applied.

Mice (*Mus musculus*) of two genotypes, db/m (20 males, 4–5 weeks old, 17.0 ± 1.1 g) and db/db (40 males, 4–5 weeks old, 21 ± 1.1 g) (Charles River, Lecco, Italy), were housed in individually ventilated cages at 22°C ± 1°C, under a 12 h circadian cycle of artificial light, with drinking water ad libitum. Upon arrival, after the quarantine period, blood glucose measurements and body weight (BW) values were used to randomize the mice into five experimental groups: SD‐fed db/m, MD‐fed db/m, SD‐fed db/db, WD‐fed db/db, and MD‐fed db/db animals. The observation period lasted 8 weeks, corresponding to a maximum age of 12 weeks for the animals. Animals were weighed weekly, with food and water consumption recorded. At sacrifice, via CO_2_ inhalation, blood and organs were collected from fasted animals and stored at −80°C for analysis. In vivo analyses were performed on all groups, while molecular and subcellular investigations focused on SD‐fed db/m, SD‐fed db/db, WD‐fed db/db, and MD‐fed db/db mice groups; MD‐fed db/m mice were excluded from this analysis subset, considering that MD in such animals did not affect any of the evaluated body and blood parameters. The WD‐fed db/db mice were sacrificed after 6 weeks due to malaise, a significant increase in blood glucose levels, and alterations in other biochemical parameters, indicating physical suffering and the risk of sudden death.

### 2.3 Analyses and Assays for Diet Characterization

To determine the fat content of the three diets, a modified fatty acid methyl esters (FAMEs) method was applied [21]. Experimental details are reported in Supporting Information. Protein content was determined following a modified Kjeldahl method [22]. Diet metabolite analysis was carried out following a modified method from Ledbetter and coworkers [23]. Experimental details are described in Supporting Information. Polyphenols were extracted and quantified following a modified method from Alonso‐Salces and colleagues; the experimental protocol is reported in Supporting Information [24]. Radical scavenging activity and reducing power of diet extracts were characterized as follows: the antiradical activity was assessed following the reduction of 2,2′‐azino‐bis‐(3‐ethylbenzothiazoline‐6‐sulfonic acid) (ABTS) radical cation in the ABTS assay [25] and of 2,2‐diphenyl‐1‐picrylhydrazyl (DPPH) in the DPPH test [26]; the ferric reducing ability power was determined by the reduction of Fe(III) to Fe(II) (FRAP assay) [27]. Further methodological details are described in Supporting Information.

### 2.4 Serum Parameters

Glucose levels were determined using the GOD‐POD colorimetric method (TRI383, Tridema). Commercially available ELISA kits were used to determine Hb1Ac (CRY‐80310, CrystalchemVinciBiochem), insulin (CRY‐90080, CrystalchemVinciBiochem), and IL‐6 (Thermo KMC0061), following the manufacturer's instructions. Serum transaminases (AST, Tridema TRI420; ALT, Tridema TRI421) and lipid profile [triglycerides (TRI447, Tridema), total cholesterol (TRI446, Tridema), and LDL (TRI246, Tridema)] were analyzed by standard procedures, according to the manufacturer's instructions. Complete blood counts were performed using an automated calibrated hematology analyzer.

### 2.5 Liver Gross Morphology and Triglyceride Content

To evaluate liver gross morphology, a photo of the livers was taken. Liver triglycerides were measured by the Mouse Triglyceride Elisa kit (MyBioSource Inc., San Diego, CA, USA) according to the manufacturer's instructions.

### 2.6 Metabolomics

Hepatic metabolite extraction was performed referring to the method of Gegner and colleagues [28], with some modifications. Liquid chromatography high‐resolution tandem mass spectrometry (LC‐MS/MS) and metabolite identification were performed using the protocols described by De Filippo and coworkers [29]. Further experimental details are described in Supporting Information.

### 2.7 Reverse Transcription (RT) Quantitative (q) PCR

RNA was isolated from the liver using the TRIZOL standard protocol (Invitrogen, California, USA). qPCR was carried out essentially as reported by Giacco and colleagues [30]. Methodological details and primer sequences used in the experiments are reported in Supporting Information and Table S1A, respectively.

### 2.8 Western Blotting

Western blotting analysis was performed as previously reported [31]. The list of the primary antibodies used in the experiments is reported in Table S1B. Protein levels were normalized to GAPDH. Proteins were detected using a chemiluminescence‐based protein‐detection method, following the protocol supplied with a commercially available kit (Millipore), and by using the appropriate secondary antibodies. Signals were quantified by means of a Bio‐Rad ChemiDoc System, using dedicated software (Image Lab 6.1, Bio‐Rad Laboratories, California, USA).

### 2.9 Separation of Respiratory Complexes by Blue‐Native PAGE and Histochemical Staining for In‐Gel Activity

Liver fragments were homogenized in ice‐cold isolation buffer (220 mM mannitol, 70 mM sucrose, 20 mM Tris‐HCl, 1 mM EDTA, 5 mM EGTA, pH 7.4). The homogenate was centrifuged at 500 × *g* for 10 min and then at 3000 × *g* for 10 min to isolate the mitochondrial pellet. Mitochondrial membranes were solubilized with 10% w/v dodecyl‐maltoside and separated by BN‐PAGE on 6%–13% T gradient gels [32]. Enzymatic activity staining for complexes I, II, and IV was performed using NADH/NTB, sodium succinate/NTB, and cytochrome c/DAB reactions, respectively [33]. Each BN‐PAGE analysis was repeated independently at least three times. For each run, parallel Coomassie staining of total protein was used as loading control. Further experimental details are reported in Supporting Information.

### 2.10 Determination of the Relative DNA Mitochondrial Copy Number

Genomic DNA was extracted and purified from approximately 20 mg of frozen liver using QIAGEN Genomic‐tip 20/G and Genomic DNA Buffer Set (Qiagen, Venlo, The Netherlands). The mitochondrial DNA (mtDNA) content was measured by real‐time PCR using a QuantStudio 5 System (Thermo Fisher Scientific, Waltham, Massachusetts, USA) essentially as reported in [30, 34]. Further experimental details are reported in Supporting Information.

### 2.11 Detection of Tissue H_2_O_2_, Carbonylated Proteins, and Lipid Peroxides

According to the manufacturer's instructions, liver endogenous H_2_O_2_ levels were measured by using the hydrogen peroxide colorimetric assay kit (Abcam, Cambridge, UK). Lipid peroxides were assayed with the Lipid Peroxidation Assay kit (Sigma‐Aldrich, Merck, Darmstadt, Germany). Carbonylated proteins were measured with the OxyBlot protein oxidation detection kit (Chemicon, Billerica, MA, USA).

### 2.12 Statistical Analyses

Statistical analyses were performed using GraphPad Prism 5 (GraphPad Software). For comparisons between two groups, an unpaired two‐tailed Student's *t*‐test was used. For comparisons among multiple groups, one‐way ANOVA followed by the Student–Newman–Keuls post hoc test was applied. Results were further validated using nonparametric tests: the Kruskal–Wallis test for multiple‐group comparisons and the Mann–Whitney *U* test for pairwise comparisons. Data are expressed as mean ± standard deviation (SD). A *p* value ≤ 0.05 was considered statistically significant.

## 3 Results

### 3.1 The MD Cocktail Is Naturally Rich in Polyphenols, Fructose, and Monounsaturated Fatty Acids

**FIGURE 1:**
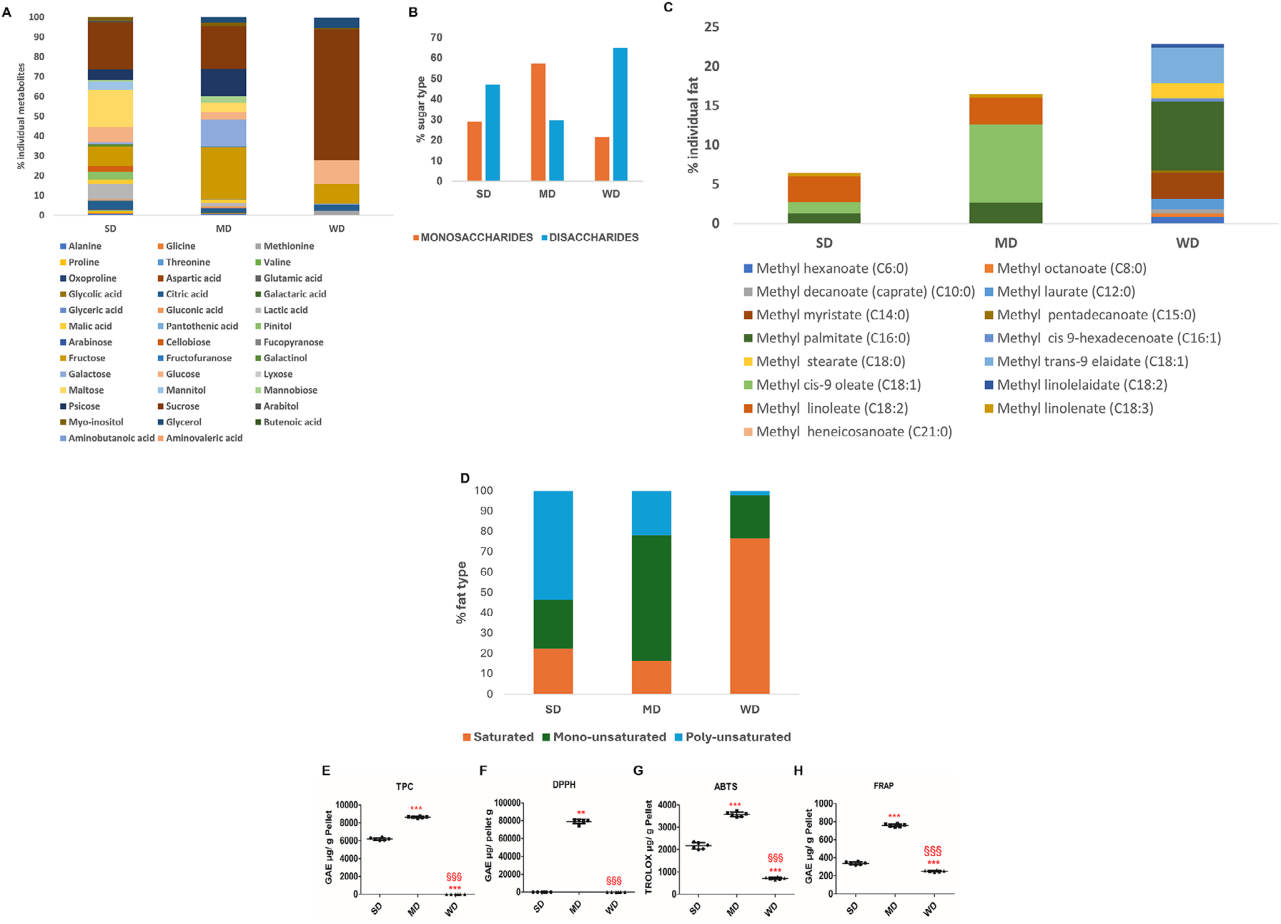
Relative concentration of metabolites and fatty acids, polyphenol content, and antioxidant activity of tested diets. (A) Metabolite analysis: graphic list of identified methanol‐extracted metabolites: sugars, amino acids, and organic acids; the height of the bar represents the percentage portion of individual metabolite in each diet. (B) Percentage composition of monomeric and dimeric sugars. (C) FAMES analysis: graphic list of identified hexane‐extracted fatty acids; the height of the bar represents the percentage portion of individual fatty acids in each diet. (D) Percentage representation of the main classes of fatty acids in each diet: SFA, MUFA, and PUFA. (E) Total polyphenol content by Folin‐Ciocalteu assay (TPC). Antioxidant activity of methanol‐ and water‐soluble metabolites was determined by (F) DPPH, (G) ABTS, and (H) FRAP assays. For experimental details, see the Materials and Methods section and Supporting Information. Data shown are mean values ± SD. One‐way ANOVA, ^**^ *p* < 0.01, ^***^ *p* < 0.001 versus SD; §§§*p* < 0.001 versus MD.

Bromatological analysis of the three diets confirmed their expected composition in macronutrients (as reported in the Materials and Methods section). GC‐MS‐based analysis revealed that carbohydrates were the most abundant methanol‐extracted metabolites: 76% in SD, 87% in MD, and 86% in WD. Other identified compounds included organic acids, amino acids, and polyalcohols, such as myo‐inositol and glycerol (Figure 1A). The main carbohydrates in SD were sucrose (24%), maltose (19%), and fructose (10%). In MD, consistently with the presence of apples in the dietary mix, fructose (26%) and sucrose (22%) dominated. WD was mostly composed of sucrose (66%) (Figure 1A). Regarding sugar proportions, disaccharides were dominant in SD (47%) over monosaccharides (29%), whereas monosaccharides prevailed in MD (57%); finally, WD had the highest disaccharide content (66%) (Figure 1B). FAMEs analysis revealed a distinct fat composition among the three diets (Figure 1C,D). SD was rich in polyunsaturated fatty acids (PUFAs) [54%, mainly methyl linoleate (C18:2/3)], with monounsaturated fatty acids (MUFAs) and saturated fatty acids (SFAs) at 24% and 23%, respectively. MD, enriched with EVOO, had a high MUFAs content (62%), with PUFAs and SFAs at 22% and 16%, respectively. Methyl cis‐9 oleate (C18:1) (60%) was the dominant MUFA, followed by methyl linoleate (C18:2/3) (21%), methyl palmitate (C16:0) (16%), and methyl linolenate (C18:3) (3%). WD was primarily composed of SFAs (77%), with methyl palmitate (C16:0) (38%) and methyl myristate (C14:0) (15%) being the major ones (Figure 1C,D). The three diets differed significantly in polyphenols and antioxidant power. Total concentration of polyphenols was undetectable in WD; in MD, this parameter was 29% higher than in SD (Figure 1E). DPPH radical scavenging activity was only detected in MD (Figure 1F). All diets showed ABTS radical and ferric reducing power, with MD having the highest values. For ABTS, MD showed a value that was 40% and 80% higher than in SD and WD, respectively; for FRAP, MD showed a value that was 56% and 67% higher than that in SD and WD, respectively (Figure 1G,H).

### 3.2 The MD Cocktail Is Neither Obesogenic nor Diabetogenic in db/M Mice and Not Hyperlipidemic in db/db Ones

**FIGURE 2:**
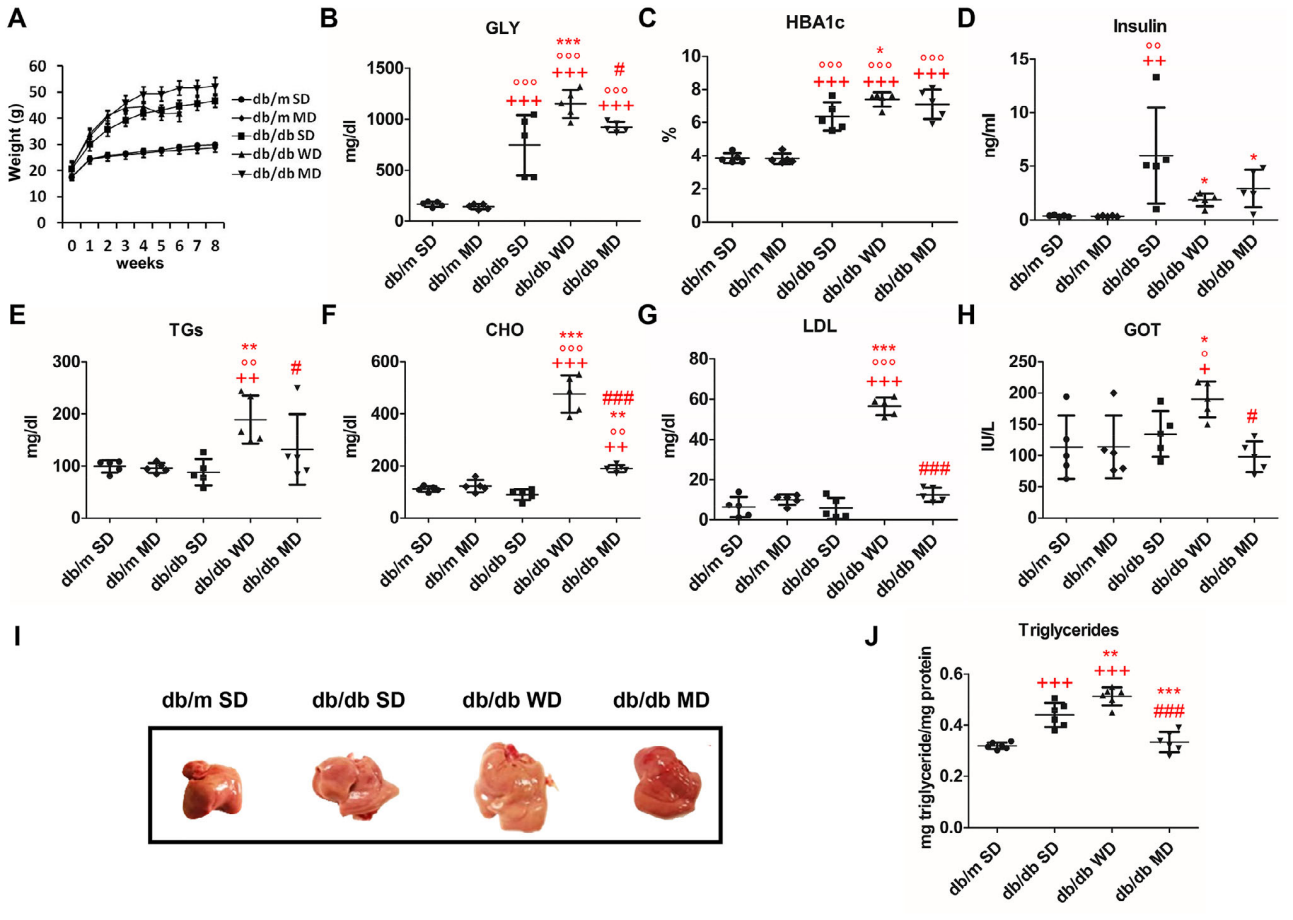
Effects of tested diets on body weight, glycemic, and lipid profiles of db/m and db/db mice. (A) Body weight; (B) glycemia (GLY); (C) glycated hemoglobin (HBA1c) percentage; (D) insulin content; (E) serum triglycerides (TGs) content; (F) total cholesterol (CHO) content; (G) LDL‐cholesterol (LDL) content; (H) glutamate oxaloacetate transaminase (GOT) level; (I) representative photographs of livers; (J) liver triglyceride content. Shown are the mean values ± SD. *n* = 5/12. One‐way ANOVA, +*p* < 0.05, ++*p* < 0.01, +++*p* < 0.001 versus SD‐fed db/m mice; °*p* < 0.05, °°*p* < 0.01, °°°*p* < 0.001 versus MD‐fed db/m mice; ^*^ *p* < 0.05, ^**^ *p* < 0.01, ^***^ *p* < 0.001 versus SD‐fed db/db mice; #*p* < 0.05, ###*p* < 0.001 versus WD‐fed db/db mice.

The body weight of control MD‐fed db/m mice was not statistically different from that of SD‐fed ones (Figure 2A), while an increased consumption of food was observed (data not shown). Compared to db/m mice, db/db diabetic mice, regardless of diet, all showed hyperphagia and a greater BW gain (Table 1; Figure 2A). During the first 3 weeks of observation, MD‐ and WD‐fed db/db mice showed completely overlapping growth curves. However, immediately afterward, while MD‐fed db/db mice regularly grew, reaching a stabilization of BW after 5 weeks of treatment, WD‐fed ones showed a decrease in BW and food intake; this phenomenon paralleled a worsening of physical conditions and blood parameters incompatible with mice survival, a condition which led to forced animal sacrifice at Week 6 (Figure 2A–H). Among db/db mice experiencing diverse diets, despite having the greatest food and energy intake, those fed the MD showed a BW gain that was statistically different only from the counterparts fed the WD (Table 1). Despite the lower BW at sacrifice, WD‐fed db/db mice showed the highest liver/BW (liver index) and mesenteric WAT/BW percentage ratios in parallel with a drastic loss of skeletal muscle mass (Table 1). Among both db/m and db/db mice, MD‐fed animals exhibited the lowest energy efficiency of body weight gain (EE‐BW) [calculated as BW gained, in mg, divided by energy intake in J, (EE‐BW)], reaching statistical significance only in control mice (Table 1). Regardless of diet and as expected, fasting glucose (GLY), glycated hemoglobin (HBA1c), and insulin levels were higher in db/db diabetic mice than db/m controls; in the latter animals, MD feeding did not produce alteration of glucose homeostasis. WD‐fed db/db mice showed the highest fasting glucose levels, a sign of severe and lethal diabetes. Even without any improvements in the diabetic profile of db/db animals, the experimental MD remained compatible with the survival of such animals that also maintained generally stable conditions (Figure 2B–D). This was not the case with WD‐fed db/db mice. The latter animals also showed severe hypertriglyceridemia, hypercholesterolemia, and high levels of glutamate oxaloacetate transaminase (GOT, also known as aspartate aminotransferase, AST) (Figure 2E–H). On the other hand, no significant differences between the groups were measured relative to blood parameter values (Figure S1) and serum levels of IL‐6 (data not shown).

**TABLE 1:** Effects of tested diets on body weight gain, tissue weight, food and energy intake, and energy efficiency of body weight gain of db/m and db/db mice.

	SD‐fed db/m	MD‐fed db/m	SD‐fed db/db	MD‐fed db/db	WD‐fed db/db
Body weight gain (g)	8.92 ± 1.07	9.82 ± 1.19	23.27 ±7.67^+++°°°^	26.85 ± 4.72^+++°°°##^	18.59 ± 2.19^++°°^
Food intake (g/week)	29.5 ± 0.95	44.25 ± 7.96^++^	42.75 ± 2.29^+++^	57.25 ± 12.99^+++°°**###^	33.75 ± 10.96
Energy intake (kJ/week)	481.44 ± 15.45	740.75 ± 133^++^	697.68 ± 37.46^+++^	958.36 ± 217^+++°°**###^	635 ± 206
Energy efficiency of body weight gain (mg/J)	2.32 ± 0.30	1.66 ± 0.35^+^	4.17 ± 1.42°	3.50 ± 0.99°	4.88 ± 1.65°
*Tissue weight*					
Liver (g)	1.22 ± 0.06	1.18 ± 0.08	2.37 ± 0.42°°°^+++^	2.72 ± 0.39°°°^+++##^	3.27 ± 0.31°°°^+++***^
Mesenteric WAT (g)	0.24 ± 0.07	0.22 ± 0.06	1.19 ± 0.29°°°^+++^	1.30 ± 0.23°°°^+++^	1.50 ± 0.29°°°^+++^
Gastrocnemius muscle (mg)	746.08 ± 132.51	893.86 ± 60.01	988.63 ± 185.98^+^	809.50 ± 271.17^###^	163.20 ± 52.96^***°°°+++^
*Tissue/Body weight*					
Liver/BW (%)	4.56 ± 0.25	4.34 ± 0.31	5.38 ± 0.66^°°°++^	5.64 ± 0.36^°°°+++###^	8.31 ± 0.77^°°°+++***^
Mesenteric WAT/BW (%)	0.89 ± 0.25	0.8 ± 0.18	2.66 ± 0.30^°°°+++^	2.7 ± 0.35^°°°+++###^	3.79 ± 0.68^°°°+++***^
Gastrocnemius muscle (%×1000)	2790.88 ± 433.84	3292.42 ± 202.15^++^	2232.97 ± 270.14^°°°++^	1671.1 ± 494.93^°°°+++###**^	408.57 ± 119.70^°°°+++***^

*Note*: Shown are the mean values ± SD. *n* = 4–8/group. One‐way ANOVA, ^++^ *p* < 0.01, ^+++^ *p* < 0.001 versus SD‐fed db/m mice; °°*p* < 0.01, °°°*p* < 0.001 versus MD‐fed db/m mice; ^**^ *p* < 0.01, ^***^ *p* < 0.001 versus SD‐fed db/db mice; ^###^ *p* < 0.001 versus WD‐fed db/db mice.

### 3.3 The MD Cocktail Affects Lipid Metabolism in the Liver of db/db Mice

**FIGURE 3:**
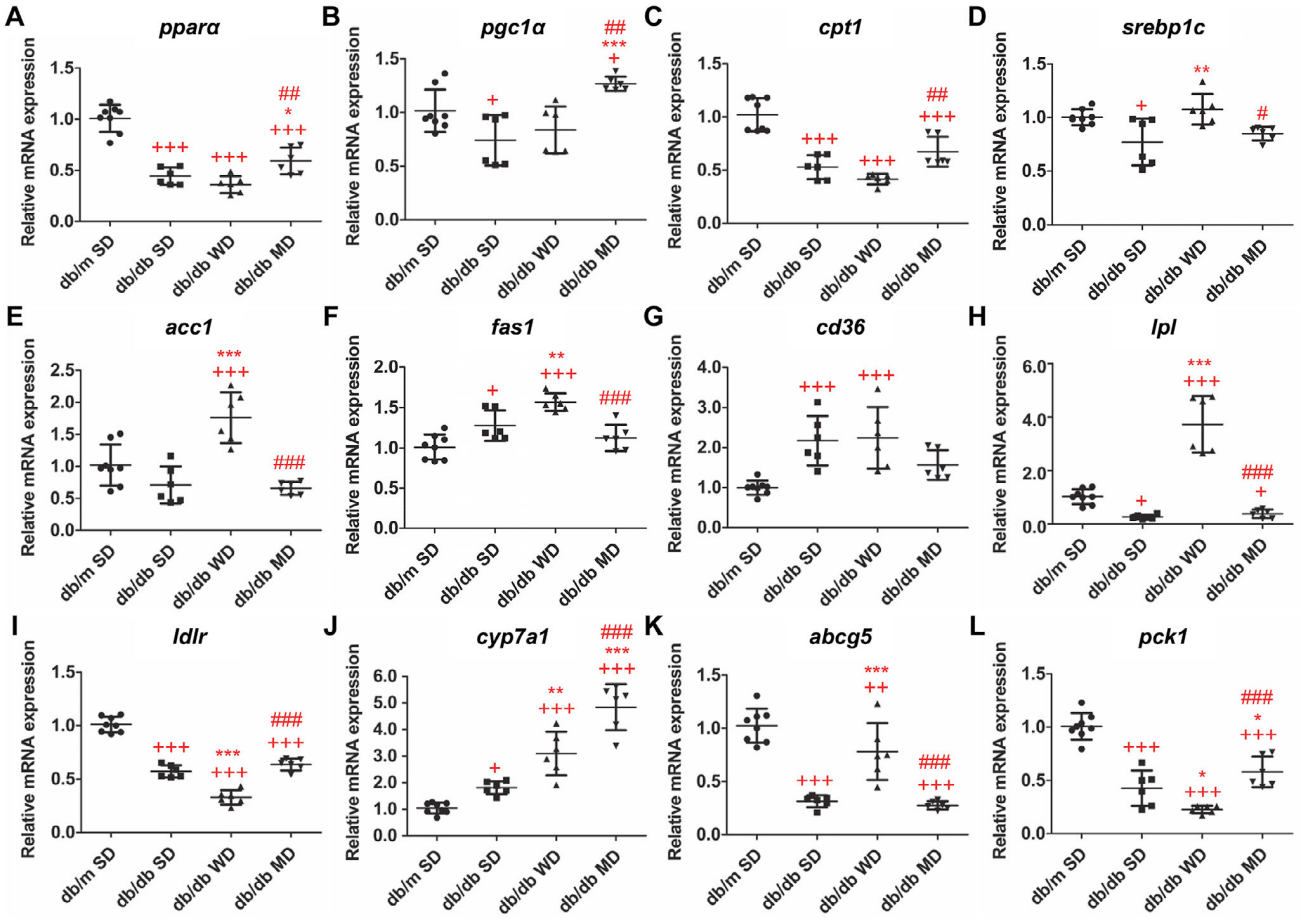
Effects of tested diets on hepatic lipid and glucose metabolism of db/m and db/db mice. mRNA expression levels of genes involved in lipolysis: (A) *pparα*; (B) *pgc1α*; (C) *cpt1*; lipogenesis: (D) *srebp1c*; (E) *acc1*; (F) *fas1*; fatty acid uptake: (G) *cd36*; lipid turnover: (H) *lpl*; cholesterol clearance: (I) *ldlr*; bile acid synthesis: (J) *cyp7a1*; bile acid excretion: (K) *abcg5*; gluconeogenesis: (L) *pck1*. Data were normalized to the values obtained for SD‐fed db/m mice (control) (set as 1); shown are the mean values ± SD. *n* = 6/8. One‐way ANOVA, +*p* < 0.05, ++*p* < 0.01, +++*p* < 0.001 versus SD‐fed db/m mice; ^*^ *p* < 0.05, ^**^ *p* < 0.01, ^***^ *p* < 0.001 versus SD‐fed db/db mice; #*p* < 0.05, ##*p* < 0.01, ###*p* < 0.001 versus WD‐fed db/db mice.

In line with data concerning the liver index (Table 1), livers from db/db mice appeared larger in size compared to those from db/m ones; livers from WD‐fed db/db mice were the largest ones (Figure 2I). In the case of SD‐ and WD‐fed db/db mice, livers appeared yellowish or whitish, while those from MD‐fed db/db animals retained a reddish color. In parallel, SD‐fed db/db mice showed significantly elevated liver TGs compared to SD‐fed db/m and MD‐fed db/db ones, a condition that was exacerbated in WD db/db counterparts (Figure 2J). In general, livers of db/db mice showed an overall downregulation of catabolic genes (*pparα* and *cpt1*) compared to that of control mice. Among db/db mice, MD‐fed ones showed significantly increased expression of *cpt1* compared to the WD‐fed group, and of *pparα* and *pgc1α* compared to both SD‐ and WD‐fed groups (Figure 3A–C). Regarding genes involved in de novo lipogenesis, such as *pparγ*, *srebp1c*, carbohydrate response element binding protein 1 (*chrebp*1), acetyl‐CoA carboxylase (*acc1*), and fatty acid synthase 1 (*fas1*), as well as those involved in triacylglycerol synthesis, including diacylglycerol o‐acyltransferase 1 and 2 (*dgat1* and *dgat2*), a differential regulation was observed between the different experimental groups (Figure 3D–F and Figure S2). In fact, *chrebp1*, *dgat1*, and *dgat2* were downregulated in the liver of all db/db mice; conversely, *pparγ* was significantly downregulated only in db/db mice consuming WD and MD, while *srebp1c* was significantly downregulated only in SD‐fed db/db mice (Figure 3D). *Fas1* was upregulated in the liver of db/db mice feeding SD and WD, with the latter animals also showing significantly augmented expression of *acc1* (Figure 3E–F). Consistently, the liver of WD‐fed db/db mice exhibited increased mRNA expression of genes involved in lipid uptake (e.g., cluster of differentiation 36, *cd36*) and lipid turnover (e.g., lipoprotein lipase, *lpl*) (Figure 3G–H). *Cd36* was also upregulated in SD‐fed db/db mice, compared to SD‐fed db/m ones. Conversely, *lpl* expression was downregulated in db/db mice consuming both SD and MD, compared to db/m SD ones.

As for cholesterol homeostasis, key genes include LDL receptor (*ldlr*), 3‐hydroxy‐3‐methylglutaryl‐CoA reductase (*hmgcr*), the direct hmgcr‐upstream regulator *srebp2*, cholesterol 7 alpha‐hydroxylase (also known as cholesterol 7‐alpha‐monooxygenase or cytochrome P450 7A1, *cyp7a1*), and ATP binding cassette subfamily G member 5 (*abcg5*). Independently of diet nature, hepatic mRNA expression of *ldlr*, *srebp2*, and *hmgcr* was significantly downregulated in all db/db mice, with WD‐fed ones showing the lowest levels (Figure 3I and Figure S2). *Cyp7a1* expression was significantly upregulated in the liver of all db/db mice, with the highest levels observed in animals consuming MD (Figure 3J). Conversely, *abcg5* expression was significantly downregulated in all db/db mice compared to SD‐fed db/m ones, showing the lowest changes in WD ones (Figure 3K). Phosphoenolpyruvate carboxykinase (*pck1*) is a multifunctional enzyme catalyzing a rate‐limiting step in gluconeogenesis and playing crucial roles in cataplerosis and glycerol‐neogenesis. Hepatic *pck1* expression was significantly downregulated in all db/db mice, independently of the diet, with the lowest and the highest values observed in WD‐ and MD‐fed animals, respectively (Figure 3L). All db/db mice also exhibited significantly reduced mRNA expression of glucose‐6‐phosphatase (*g6p*), another key enzyme regulating hepatic glycogenolysis (Figure S2).

### 3.4 The MD Cocktail Improves Oxidative Balance in the Liver of db/db Mice

**FIGURE 4:**
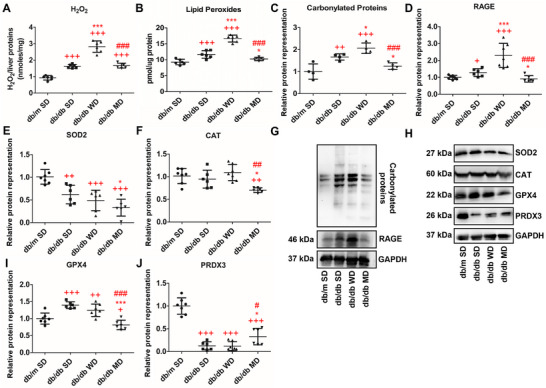
Oxidative balance and damage in the liver of db/m and db/db mice fed different diets. Liver content of oxidative stress markers (A) H_2_O_2_ and (B) lipid peroxides. (C–F, I, and J) Quantitative analysis and (G and H) representative western blots of other oxidative damage markers: carbonylated proteins and RAGE, and key antioxidant enzymes: SOD2, CAT, GPX4, and PRDX3. Data were normalized to the values obtained for SD‐fed db/m mice (control) (set as 1); shown are the mean values ± SD. *n* = 4/7. One‐way ANOVA, +*p* < 0.05, ++*p* < 0.01, +++*p* < 0.001 versus SD‐fed db/m mice; ^*^ *p* < 0.05, ^**^ *p* < 0.01, ^***^ *p* < 0.001 versus SD‐fed db/db mice; #*p* < 0.05, ##*p* < 0.01, ###*p* < 0.001 versus WD‐fed db/db mice. For RAGE, *t*‐test, +*p* < 0.05 vs. SD‐fed db/m mice and ^*^ *p* < 0.05 versus SD‐fed db/db mice.

Elevated H_2_O_2_ levels were observed in the liver of SD‐fed db/db mice compared to db/m animals consuming SD (control), whereas the highest increase of this oxidant molecule was measured in the organ of WD‐fed db/db mice. H_2_O_2_ levels in MD‐fed db/db mice were higher than those in SD db/m ones, in line with SD‐fed db/db counterparts, and approximately half of those of WD‐fed db/db animals (Figure 4A). Additionally, lipid peroxides were elevated in SD‐fed db/db mice compared to SD‐fed db/m and MD‐fed db/db counterparts, whereas db/db animals consuming WD showed the highest relative concentrations (Figure 4B). Looking at the markers of oxidative damage in cellular proteins, carbonylation of liver proteins was significantly higher in SD‐ and WD‐fed db/db mice, compared to SD‐fed db/m and MD‐fed db/db animals, with no differences between the latter ones (Figure 4C,G). Protein levels of receptors of advanced glycation end‐products (RAGE), another indicator of altered liver metabolism and oxidative stress, were unchanged in MD‐fed db/db mice versus control, slightly but significantly elevated in SD‐fed db/db ones, and markedly higher in WD db/db‐fed counterparts (Figure 4D,G). As for antioxidant enzymes, superoxide dismutase 2 (SOD2) content was lower in all db/db mice groups compared to SD db/m ones, with the lowest mean value in MD‐fed db/db mice (Figure 4E,H). Catalase (CAT) representation in the liver was significantly lower in MD‐fed db/db mice compared to all the other groups (Figure 4F,H). Lastly, glutathione peroxidase 4 (GPX4) and peroxiredoxin‐3 (PRDX3) showed opposite trends (Figure 4H–J); GPX4 increased in SD‐ and WD‐fed db/db mice, whereas PRDX3 decreased in the above‐reported animals. MD‐fed db/db mice showed reduced GPX4 levels with respect to all the other animal groups, especially the diabetic ones, and a higher PRDX3 content compared to SD‐ and WD‐fed db/db ones.

### 3.5 The MD Cocktail Ameliorates Mitochondrial Biogenesis and Dynamics in the Liver of db/db Mice

**FIGURE 5:**
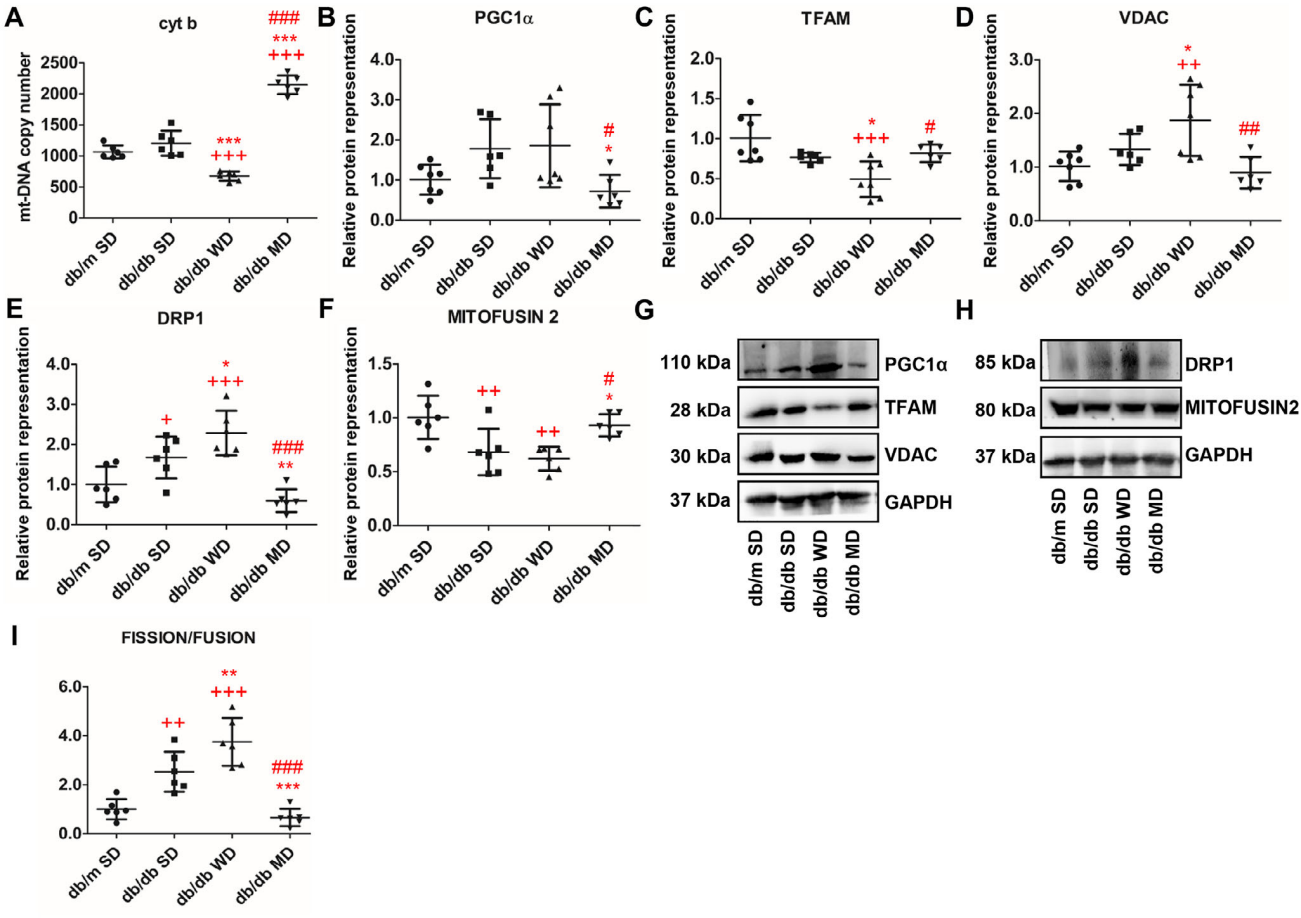
Mitochondrial biogenesis and dynamics in the liver of db/m and db/db mice fed different diets. (A) Relative mtDNA copy number, a marker of mitochondrial mass. Shown are the mean values ± SD. (B–F) Relative representation level and (G–H) representative western blots of protein involved in biogenesis (PGC1α, TFAM, and VDAC) and mitochondrial dynamics (DRP1 and MITOFUSIN 2). (I) Fission/fusion ratio. Data were normalized to the values obtained for SD‐fed db/m mice (control) (set as 1); shown are the mean values ± SD. *n* = 6/8. One‐way ANOVA, +*p* < 0.05, ++*p* < 0.01, +++*p* < 0.001 versus SD‐fed db/m mice; ^*^ *p* < 0.05, ^**^ *p* < 0.01, ^***^ *p* < 0.001 versus SD‐fed db/db mice; #*p* < 0.05, ##*p* < 0.01, ###*p* < 0.001 versus WD‐fed db/db mice.

**FIGURE 6:**
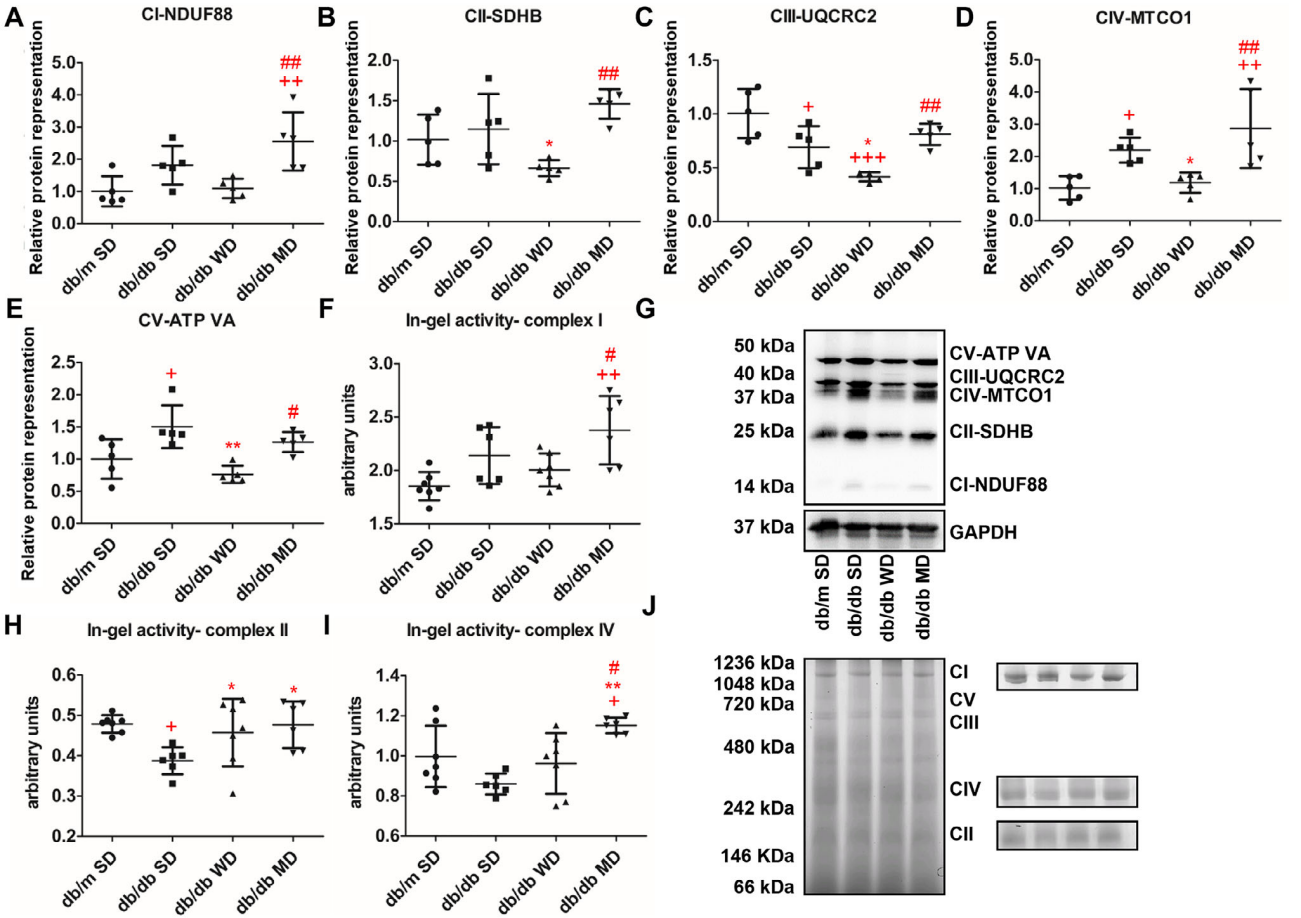
Effects of tested diets on liver mitochondrial OXPHOS of db/m and db/db mice. (A–E) Relative representation and (G) representative western blots of the five subunits of OXPHOS. Data were normalized to the values obtained for SD‐fed db/m mice (control) (set as 1); shown are the mean values ± SD. (F, H, I) Densitometric quantification of bands corresponding to individual in‐gel activity of complexes I, II, and IV. (J) Representative image of a BN‐PAGE gel and histochemical staining of complex I (CI), complex IV (CIV), and complex II (CII) in‐gel activity. Data were reported as relative intensity and presented separately for each treatment (mean values ± SD). *n* = 5/7. One‐way ANOVA, +*p* < 0.05, ++*p* < 0.01, +++*p* < 0.001 versus SD‐fed db/m mice; ^*^ *p* < 0.05, ^**^ *p* < 0.01 versus SD‐fed db/db mice; #*p* < 0.05, ##*p* < 0.01 versus WD‐fed db/db mice.

In db/db mice, differential ROS accumulation, tissue damage, and antioxidant alterations pinpointed how the three dietary regimens affect liver mitochondrial function, with a focus on markers of biogenesis and dynamics. Firstly, the relative mtDNA copy number, one of the key sensors and regulators of the energetic state and mitochondrial biogenesis, was analyzed. Representation of the mitochondrial marker gene cytochrome B (cytb) resulted significantly higher in the liver of MD‐fed db/db mice compared to all the other animal groups (+69% vs. WD‐fed db/db ones, +45% vs. SD‐fed db/db ones, and +50% vs. SD‐fed db/m ones). A specific decreased representation was observed in db/db mice consuming WD (Figure 5A). Then, we evaluated the hepatic representation of protein markers of mitochondrial biogenesis. Levels of PGC1α, very similar in SD‐fed db/m and MD‐fed db/db mice, were significantly higher in both db/db animals nourished with SD and WD (Figure 5B,G). Levels of TFAM, a final key activator of mitochondrial transcription, were diminished in the liver of WD‐fed db/db mice compared to all the other animal groups (Figure 5C,G). In contrast, levels of the mitochondrial structural protein voltage‐dependent anion channel, VDAC, were significantly overrepresented only in the liver of WD‐fed db/db mice (Figure 5D,G). When mitochondrial dynamics was evaluated, a significant increase in the representation levels of the fission protein DRP1 was observed in the liver of SD‐ and WD‐fed db/db mice compared to MD‐fed db/db (+75%) and SD‐fed db/m (+57%) counterparts; db/db mice nourished by MD were characterized by unchanged levels of DRP1 compared to SD‐fed db/m ones (Figure 5E,H). An opposite trend was observed for the fusion protein MITOFUSIN 2; while its hepatic levels significantly decreased in WD‐ and SD‐fed db/db mice, they remained unchanged in MD‐fed db/db versus SD‐fed db/m animals (Figure 5F,H). Therefore, the calculated fission/fusion ratio significantly increased in db/db mice feeding WD, compared to all the other experimental groups (+81% vs. MD‐fed db/db ones, +35% vs. SD‐fed db/db ones, and +71% vs. SD‐fed db/m ones). A less pronounced but similar quantitative trend was also observed for SD‐fed db/db mice, compared to SD‐fed db/m or MD‐fed db/db animals (Figure 5I), suggesting an unbalanced mitochondrial dynamics toward fission in the liver of SD‐ and WD‐fed db/db mice; conversely, MD feeding preserved mitochondrial dynamics in db/db mice. To evaluate the effects of the three diets on the mitochondrial OXPHOS system, the protein representation level of core subunits of the five mitochondrial respiratory complexes (CI‐V) was measured (Figure 6A–E,G). Compared to SD‐fed db/m mice, SD‐fed db/db ones showed unchanged levels of CI‐NDUF88 and CII‐SDHB, reduced levels of CIII‐UQCRC2, and increased levels of both CIV‐MTCO1 and CV‐ATP VA. On the other hand, WD‐fed db/db mice showed an overall down‐representation of all the mitochondrial respiratory chain complexes with respect to all the other db/db animal groups, reaching a statistical significance compared to SD‐fed db/m mice only in the case of CIII‐UQCRC2. Compared to SD‐fed db/m and WD‐fed db/db mice, db/db animals at MD showed significantly higher levels of CI‐NDUF88 (+57% and +57%, respectively) and CIV‐MTCO1 (+65% and +60%, respectively) (Figure 6A–E,G). Respiratory chain complexes from liver mitochondria of db/m and db/db mice experiencing a different diet were further resolved by BN‐PAGE and analyzed for their individual *in‐gel* activities (calculated as specific enzyme activity). Profiles of Coomassie blue‐stained OXPHOS complexes and *in‐gel* activities of purified complexes CI, CII, and CIV are shown in Figure 6F,H–J. Densitometric analysis revealed that MD‐fed db/db mice showed a significantly increased activity of CI and CIV when compared to SD‐fed db/m and WD‐fed db/db counterparts, whereas that of CII remained unchanged. MD‐fed db/db mice also showed increased activity of complex IV compared to db/db animals fed SD. The latter mice were in turn characterized by a significantly reduced activity of the CII complex when compared to all the other experimental groups.

### 3.6 The MD Cocktail Ameliorates Autophagy and Mitophagy in the Liver of db/db Mice

**FIGURE 7:**
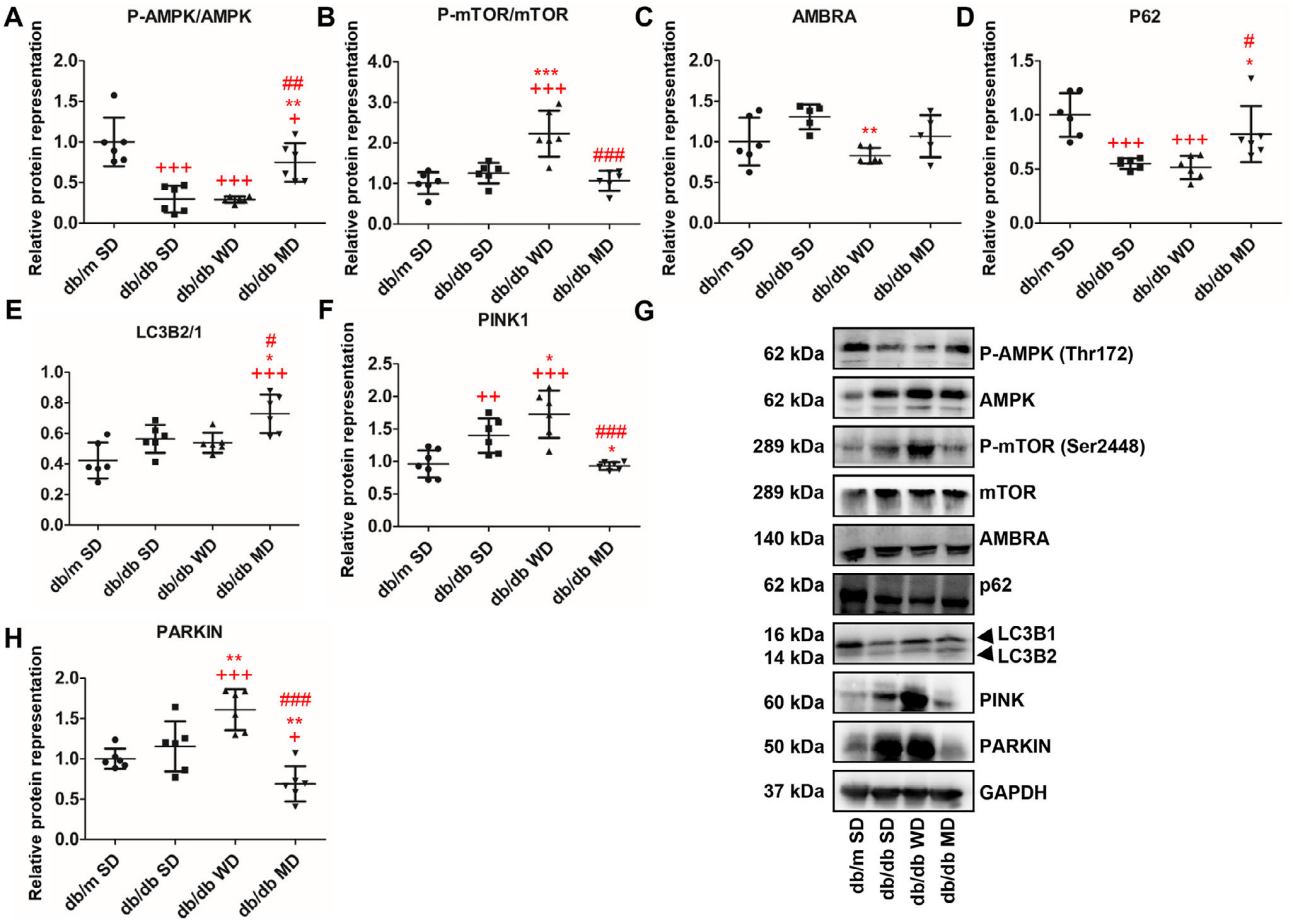
Autophagy and mitophagy in the liver of db/m and db/db mice fed different diets. (A–F, H) Quantitative analysis and (G) representative western blots of markers of autophagy (upstream regulators and key effectors: P‐AMPK/AMPK, P‐TOR/mTOR, AMBRA, P62, and LC3B2/1) and mitophagy (PINK1 and PARKIN). Data were normalized to the values obtained for SD‐fed db/m mice (control) (set as 1); shown are the mean values ± SD. *n* = 6. One‐way ANOVA, +*p* < 0.05, ++*p* < 0.01, +++*p* < 0.001 versus SD‐fed db/m mice; ^*^ *p* < 0.05, ^**^ *p* < 0.01, ^***^ *p* < 0.001 versus SD‐fed db/db mice; #*p* < 0.05, ##*p* < 0.01, ###*p* < 0.001 versus WD‐fed db/db mice.

To verify whether the three diets might influence cellular homeostatic processes such as autophagy and mitophagy, representation levels of markers for autophagy, such as AMBRA, P62, and LC3B2/1, and for mitophagy, such as PARKIN and PINK1, were evaluated together with phosphorylation analysis of two of the major upstream regulators of autophagy, namely P‐AMPK (Thr172) and P‐mTOR (Ser2448). Phosphorylation levels of Thr172‐AMPK were significantly reduced in all db/db mice, with a significant over‐representation in MD‐fed compared to WD‐ and SD‐fed db/db animals (+60% and +61%, respectively, Figure 7A,G). On the other hand, Ser2448‐mTOR phosphorylation levels were higher in db/db mice consuming WD compared to all the other experimental groups (Figure 7B,G). AMBRA, a key interacting factor to initiate the formation of autophagosomes, exhibited a significant down‐representation in WD‐fed db/db mice, compared to SD‐fed db/db animals (Figure 7C,G). As for P62, essential for its ability to bind the ubiquitinated cargo and LC3B, SD‐ and WD‐fed db/db mice exhibited decreased protein expression levels (−32% and −37%, respectively) compared to db/m animals experiencing SD (Figure 7D,G). No statistically significant differences were observed when MD‐fed db/db and SD‐fed db/m mice were compared. On the other hand, the hepatic index of autophagosome formation and autophagy functioning (LC3B2/1 ratio) increased in MD‐fed db/db mice compared to all the other experimental groups (+26% vs. WD‐fed db/db ones, +23% vs. SD‐fed db/db ones, and +42% vs. SD‐fed db/m ones) (Figure 7E,G). PINK1 and PARKIN, the ones tagging damaged mitochondria with ubiquitin chains for selective autophagy, showed a similar representation trend. Both were significantly overrepresented in the liver of WD‐fed db/db mice compared to all the other groups. Furthermore, they were respectively increased or unchanged in the SD‐fed db/db versus SD‐fed db/m animal comparison, increased in the SD‐fed db/db versus MD‐fed db/db mice resemblance, and unchanged or reduced in the MD‐fed db/db versus SD‐fed db/m mice comparison (Figure 7F–H). All together, these data suggest the occurrence of a blockage of autophagy and an activation of mitophagy in the liver of db/db mice consuming WD and SD, while a normalization of such homeostatic functions occurs in MD‐fed db/db mice.

### 3.7 The MD Cocktail Tackles Tissue Fibrosis and Hypoxia in the Liver of db/db Mice

**FIGURE 8:**
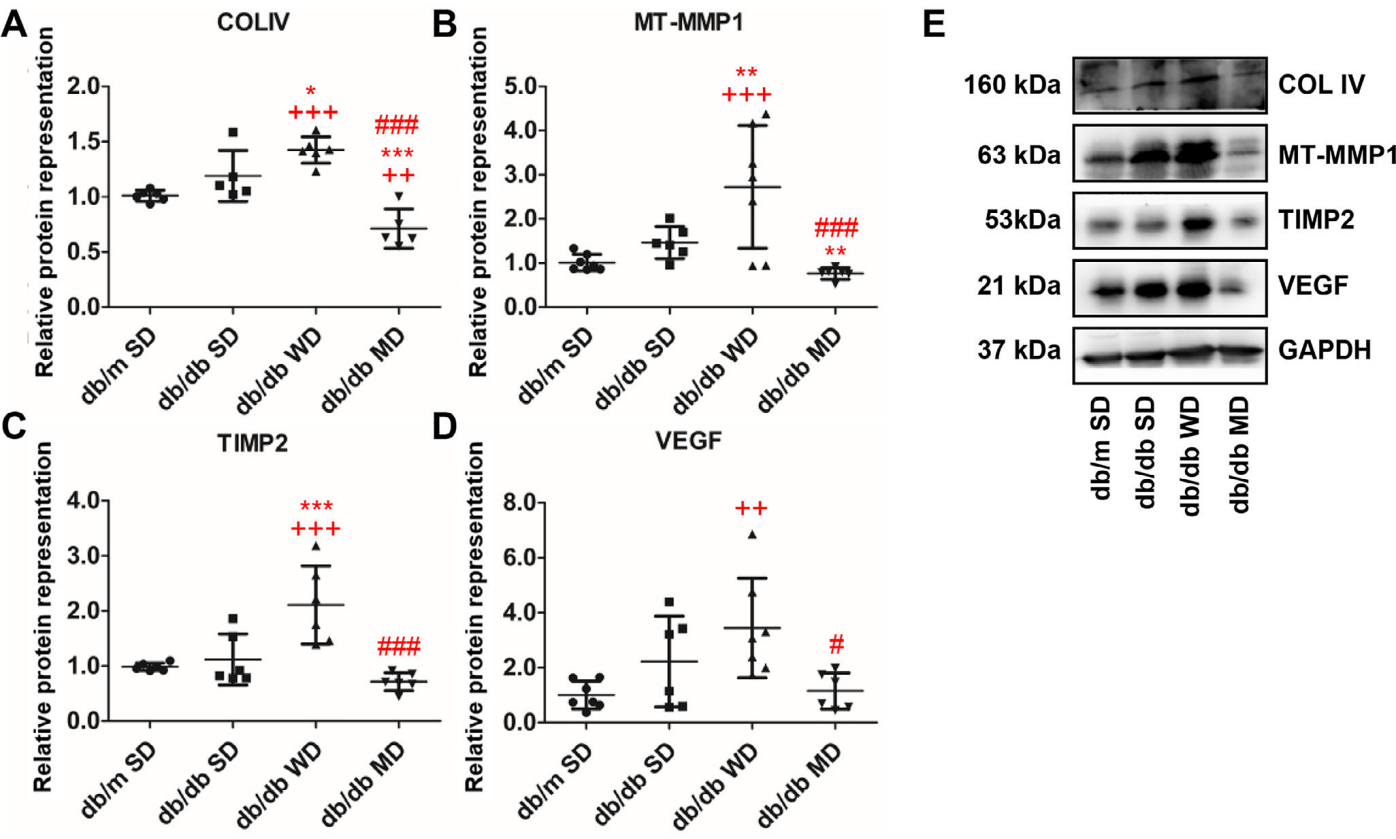
Fibrosis and hypoxia in the liver of db/m and db/db mice fed different diets. (A–D) Quantitative analysis and (E) representative western blots of markers of fibrosis (COLIV, MT‐MMP1, and TIMP2) and hypoxia (VEGF). Data were normalized to the values obtained for SD‐fed db/m mice (control) (set as 1); shown are the mean values ± SD. *n* = 5/7. One‐way ANOVA, ++*p* < 0.01, +++*p* < 0.001 versus SD‐fed db/m mice; ^*^ *p* < 0.05, ^**^ *p* < 0.01, ^***^ *p* < 0.001 versus SD‐fed db/db mice; #*p* < 0.05, ###*p* < 0.001 versus WD‐fed db/db mice. For MT‐MMP1, *t*‐test, ^**^ *p* < 0.05 versus SD‐fed db/db mice.

Since alterations of mitochondrial functions can be associated with progression to the necro‐inflammatory and fibrotic form of liver disease, the representation of markers of liver fibrosis, hypoxia, and inflammation was evaluated. Protein levels of collagen type IV (COLIV), a structural scaffold within the extracellular matrix, were significantly increased in WD‐fed db/db mice compared to all the other groups, while they significantly decreased in MD‐fed db/db animals (Figure 8A,E). Similarly, membrane‐type matrix metalloproteinases (MT‐MMP1), responsible for the degradation of extracellular matrix components, and TIMP2, a tissue inhibitor of metalloproteinase‐2, were both significantly overrepresented in WD‐fed db/db mice compared to all the other experimental groups (Figure 8B,C,E). A slight and insignificant decrease in their representation was observed when MD‐fed db/db mice were compared to SD‐nourished db/m animals. For MT‐MMP1A, a significant decrease of protein levels was observed in the MD‐fed versus SD‐fed db/db mice comparison. Finally, the vascular endothelial growth factor (VEGF), a key player in angiogenesis and a marker of hypoxia, was significantly increased in db/db mice consuming WD compared to MD‐fed db/db and SD‐fed db/m animals (Figure 8D,E).

### 3.8 The MD Cocktail Preserves Hepatic Concentration of Metabolites Essential for Energy Homeostasis and Substrate Fluxes

**FIGURE 9:**
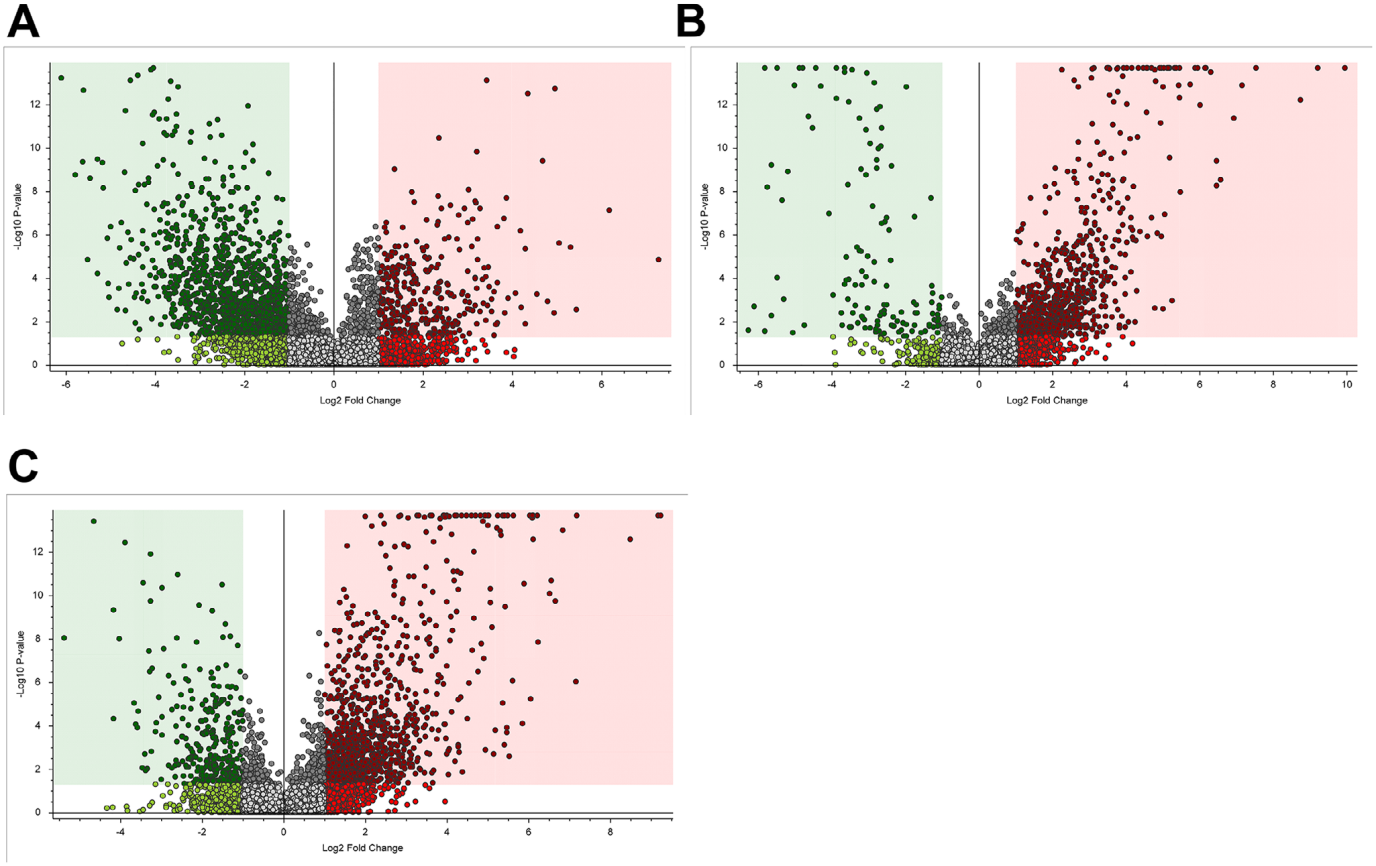
Metabolomics of liver tissues from db/m and db/db mice fed different diets. Volcano plots depict the compound area counts through a 1 versus 1 comparison in the liver upon the three different conditions representing the changed liver metabolites in the comparison: (A) SD‐fed db/m mice versus SD‐fed db/db mice, (B) MD‐fed db/db mice versus SD‐fed db/db mice, and (C) MD‐fed db/db mice versus WD‐fed db/db mice. Red full circles, over‐represented metabolites; green full circles, down‐represented metabolites; gray full circles, not significantly changed metabolites by considering Log2FoldChange: 1; *p* value < 0.05. False discovery rate correction was applied through Benjamini–Hochberg post hoc analysis, and a significance level of 0.05 was used. Starting with all the identified compounds (Table S2), we pinpointed those that significantly changed through the volcano plots; within these two groups, we quantified all the compounds with chemical behavior in line with pure analytical standards as reported in Table S3.

**FIGURE 10:**
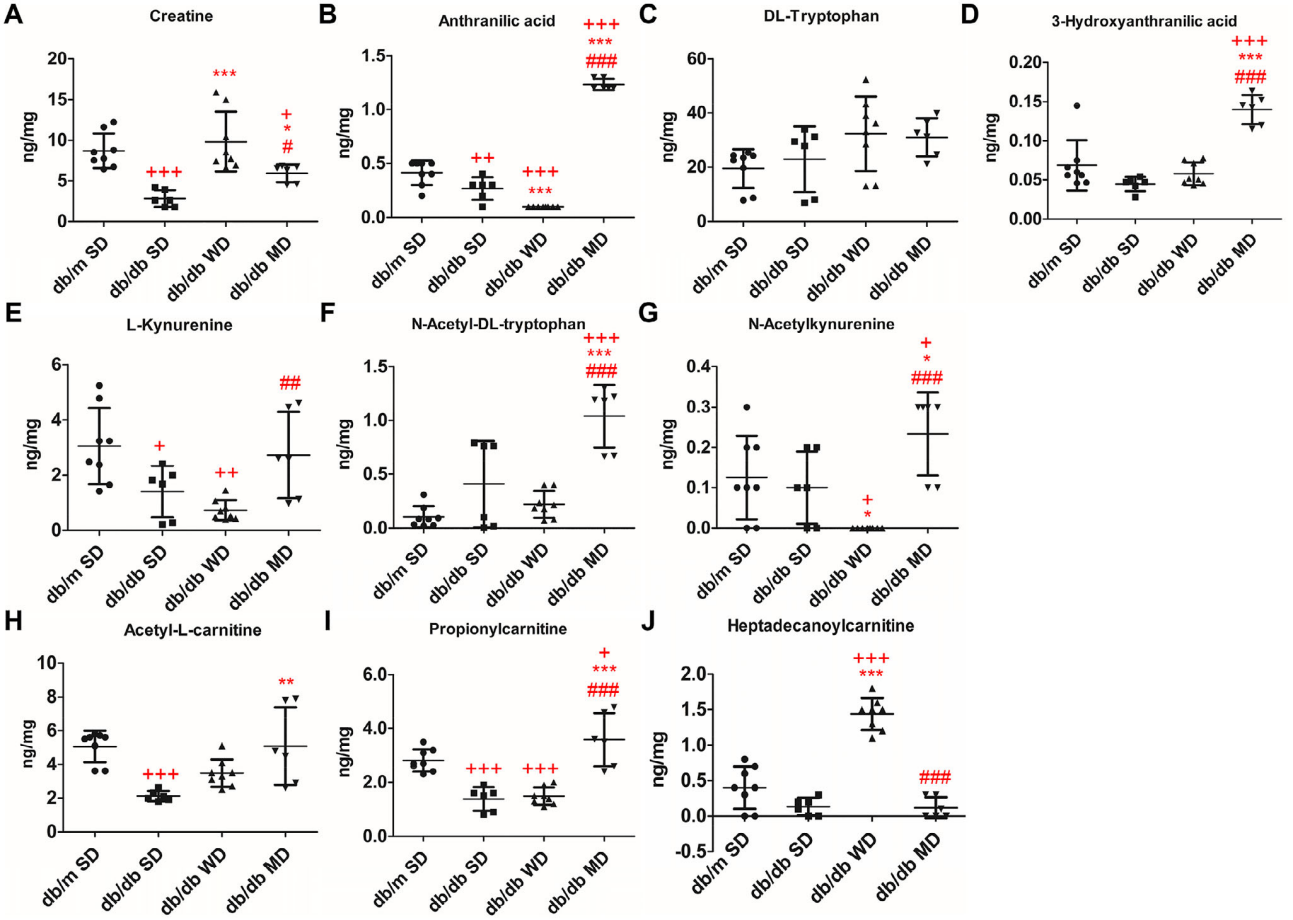
Effects of tested diets on selected liver metabolites. Quantitative analysis of (A) creatine, (B–G) tryptophan and its derivatives, and (H–J) acylated carnitine derivatives. Data are shown as mean values ± SD (ng/mg). *n* = 6/8. One‐way ANOVA, +*p* < 0.05, ++*p* < 0.01, +++*p* < 0.001 versus SD‐fed db/m mice; ^*^ *p* < 0.05, ^***^ *p* < 0.001 versus SD‐fed db/db mice; ##*p* < 0.01, ###*p* < 0.001 versus WD‐fed db/db mice.

**FIGURE 11:**
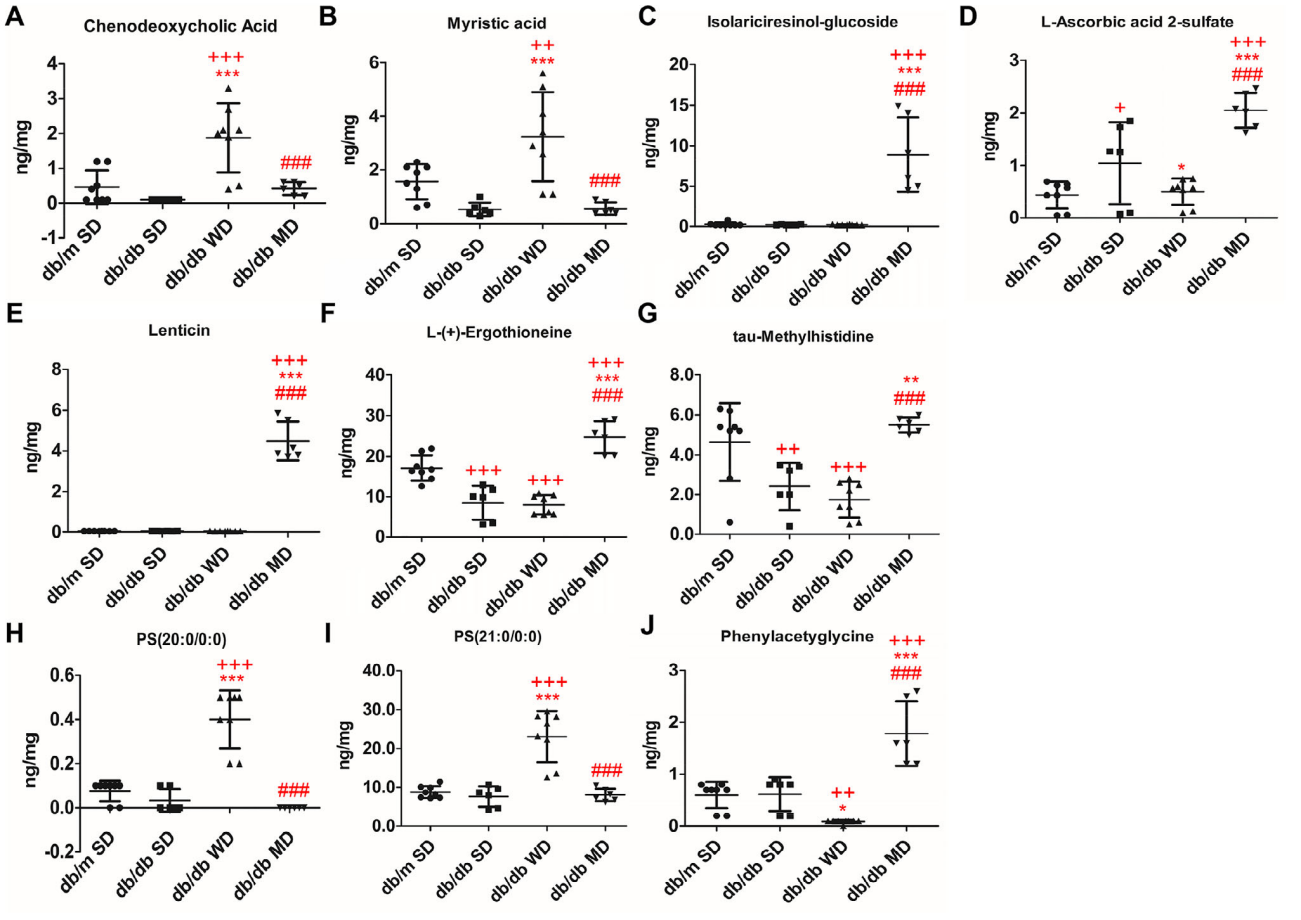
Effects of tested diets on selected liver metabolites. Quantitative analysis of (A) chenodeoxycholic acid, (B) myristic acid, (C–F) phyto‐derived antioxidants, (G) tau‐methylhistidine, (H–I) phosphatidylserine derivatives, and (J) phenylacetylglycine. Data are shown as mean values ± SD (ng/mg). *n* = 6/8. One‐way ANOVA, ++*p* < 0.01, +++*p* < 0.001 versus SD‐fed db/m mice; ^*^ *p* < 0.05, ^**^ *p* < 0.01, ^***^ *p* < 0.001 versus SD‐fed db/db mice; ###*p* < 0.001 versus WD‐fed db/db mice.

LC‐MS/MS‐based metabolomics of liver tissues of mice experiencing different diets spotted a complex molecular pattern of compounds having a mass value within the *m/z* range 80–800. The analytical workflow detected a total of 8892 common chemical features that are part of polar and nonpolar compound families (Table S2). Pairwise comparisons and discriminant analysis revealed a differential compound representation for diabetes (SD‐fed db/db mice vs. SD‐fed db/m mice), Mediterranean diet (MD‐fed vs. SD‐fed db/db mice), and Western diet (WD‐fed vs. MD‐fed db/db mice) (Figure S3). Using a stringent threshold as a ≥ 2‐fold change and *p* < 0.05, 1538 compounds showed significant concentration changes in the SD‐fed db/db mice versus SD‐fed db/m mice comparison [356 metabolites (23%) overrepresented, and 1182 metabolites (77%) down‐represented]; on the other hand, 943 compounds showed altered levels in the MD‐fed versus SD‐fed db/db mice comparison [808 metabolites (86%) overrepresented, and 135 metabolites (14%) down‐represented]; finally, 1287 compounds showed significant concentration changes in the WD‐fed versus MD‐fed db/db mice comparison [1008 metabolites (78%) overrepresented and 279 metabolites (22%) down‐represented] (Figure 9A–C). Considering the chemical nature of the compounds included in the analytical workflow, the diabetic condition resulted in an overall reduced molecular representation. According to metabolite identification and annotation, several pathways were significantly influenced by the different diets. Specifically, creatine and tryptophan metabolism pinpointed direct effects on indole rings, aromatic amino acids, and polar hydroxylated side chain amino acids (Figure 10A–G). Indeed, such precursors are intrinsically linked to the biosynthesis of crucial and functional compounds, whose deficiencies are associated with metabolic disorders. Overall, MD administration elicited a protective effect on hepatic energy and metabolic fluxes. Compared to SD‐fed db/db mice, MD‐fed counterparts showed an increased hepatic concentration of creatine, highlighting a trend toward normalization to control levels. In contrast, among diabetic mice, WD‐fed db/db animals exhibited the highest creatine levels (Figure 10A), likely reflecting hepatic accumulation of this metabolite, consistent with their reduced skeletal muscle mass. Moreover, while DL‐tryptophan levels remained unchanged, MD promoted an increase in the concentration of anthranilic acid, 3‐hydroxyanthranilic acid, N‐acetyl‐DL‐tryptophan, and N‐acetylkynurenine compared to all the other experimental groups (Figure 10B–G) and normalized levels of L‐kynurenine to control levels with respect to SD‐ and WD‐fed db/db mice. In humans, tryptophan is an essential amino acid that is vital for the production of methoxyindole and kynurenine, the latter being a key compound for NAD biosynthesis. Metabolites in such a pathway affect insulin function [35], while NAD helps to prevent diet‐induced hepatic steatosis [36]. Thus, MD may influence tryptophan metabolism in a potentially beneficial manner, although the precise effects and dose‐dependencies of individual metabolites remain to be clarified. These results suggest that MD stimulates tryptophan and NAD biosynthesis and tryptophan acetylation in the gut [37]. On the other hand, diabetic mice on SD exhibited reduced hepatic levels of short‐ and long‐chain acylated carnitine derivatives (Figure 10H–J), key players in β‐oxidation of LCFAs, mitochondrial energy production, and metabolic homeostasis. MD feeding in db/db mice restored or even augmented short‐chain acylated carnitine derivative levels (Figure 10H,I and Figure S4), suggesting a positive effect of this diet on mitochondrial β‐oxidation. Conversely, along with reduced levels of short‐chain acylated carnitine derivatives (Figure 10H,I), WD‐fed db/db mice showed significantly elevated levels of long‐chain acylated carnitines (Figure 10J), which have already been associated with a blockage of mitochondrial β‐oxidation. Cholesterol homeostasis is tightly regulated by primary bile acid biosynthesis, which plays a central role in maintaining hepatic lipid balance. Among diabetic animals, WD‐fed db/db mice, paralleling their hepatic gene expression and systemic hypercholesterolemia, were the only ones showing the highest concentration in the liver of chenodeoxycholic acid, together with an accumulation of saturated fatty acids as myristic acid, saturated phosphatidylserine derivatives, and maltose (Figure 11A,B,H,I; Figure S4); this phenomenon was not observed in SD‐ and MD‐fed db/db mice. Notably, db/db animals consuming MD uniquely accumulated phyto‐derived antioxidants, including dietary polyphenols such as isolariciresinol (Figure 11C), vitamins (i.e., L‐ascorbic acid 2‐sulfate, Figure 11D), lenticin (Figure 11E), and ergothioneine (Figure 11F), with the latter imidazole derivative acting as a powerful scavenger of hydroxyl radicals. Other metabolites showed a diet‐specific hepatic enrichment: tau‐methylhistidine was significantly reduced in SD‐ and WD‐fed db/db mice and normalized in MD‐fed db/db animals (Figure 11J), reflecting the daily intake of cod and chicken [38]; phenylacetylglycine, resulting from microbiota metabolism of polyphenols in the rodent gut, was augmented in MD‐fed db/db mice, compared to all the other experimental groups (Figure 11G).

## 4 Discussion

In this study, an experimental diet was developed to mimic the composition of the traditional MD, and it was compared with SD and WD for its consequences after feeding db/m and db/db mouse models. After 8 weeks of intervention, db/db mice fed MD, featuring high levels of polyphenols, MUFAs (e.g., oleate), and monosaccharides (e.g., fructose), and showing great antioxidant activity, displayed distinct metabolic responses; while exhibiting higher caloric intake and greater weight gain than SD‐fed counterparts, they maintained a lower hepatic index than WD‐fed animals, which exhibited pronounced metabolic derangements. On the other hand, WD‐fed db/db mice showed higher mesenteric fat/body weight ratios, significant loss of skeletal muscle mass, and impaired survival, an overall severe conditionthat necessitated their ethical sacrifice at 6 weeks. This introduced a time‐point discrepancy with other groups (sacrificed at 8 weeks), which may have influenced the comparability of disease progression and treatment effects. However, the observed accelerated deterioration was considered an indicator of WD's severity, and results were interpreted accordingly. Hepatic and adiposity indexes of MD‐fed db/db mice were not significantly different from those of SD‐fed ones, which, however, had a 27% lower energy intake and consumed a standard low‐fat chow (−60% fat intake compared to db/db mice consuming MD). This suggests that, despite higher fat and calories, the MD did not worsen adiposity or liver weight, possibly due to better metabolic handling of nutrients. MD‐fed mice also showed on average a lower energy efficiency than both SD and WD groups, pointing to a reduced conversion of calories into fat, such a parameter being statistically significant in control animals, likely reflecting the impact of nutrient quality on energy use and fat storage. While MD‐fed db/db mice were not protected from hyperglycemia and weight gain, they showed better physiological resilience compared to WD‐fed db/db animals, which suffered from severe hyperglycemia, dyslipidemia, hepatic steatosis, and liver dysfunction by Week 6. The MD‐fed db/db mice maintained lower hepatic triglycerides and demonstrated improved lipid profiles, suggesting a partial protective effect on systemic metabolism. These results underscore the MD potential to modulate metabolic outcomes and preserve survival in the presence of diabetes and obesity, although it cannot fully counteract the disease‐driven metabolic dysfunctions likely due to persistent defects in glucose homeostasis (e.g., MD did not counteract diabetes‐induced alterations in GLUT2 and AKT protein levels; data not shown) and/or pancreatic function (not analyzed).

MD significantly influenced liver function and gene expression profiles, particularly pathways involved in lipid metabolism, mitochondrial function, and oxidative stress. Upregulation of key hepatic genes, such as *pgc1α*, *cyp7a1*, and *pck1*, in MD‐fed db/db mice suggested enhanced mitochondrial oxidative capacity, improved bile acid metabolism, and active cataplerosis. These changes are consistent with more efficient lipid clearance and cholesterol excretion in MD‐fed db/db animals, evidenced by their favorable blood lipid profiles and lower liver triglyceride accumulation compared to WD‐fed counterparts. *Pgc1α* is considered a functional target of the EVOO secoiridoid‐related polyphenols, such as, among others, oleuropein and hydroxytyrosol [39]. *Pgc1α* upregulation was described to improve mitochondrial function, resulting in an increasing COX activity [41], while its knockdown was reported to abolish hydroxytyrosol‐improved mitochondrial activity [42]. In the present study, we observed a significant MD‐associated stimulation of *pgc1α* transcription with its protein expression fixed at healthy levels. This apparent discrepancy might be rationalized by considering that at the time point of analysis the mtDNA copy number, and thus mitochondrial mass, appeared significantly enhanced, in line with the compensated blood lipid profile. Interestingly, EVOO metabolites as phenylacetic derivatives putatively arising from the gut microbiota were significantly over‐represented in the liver of MD‐fed db/db mice compared to WD‐fed ones. This distribution pinpointed an interconnection between gut‐metabolites and liver catabolites [40]. Quercetin, abundantly present in onions, can be considered a good candidate as an MD mediator of the observed *cyp7a1*‐associated and chenodeoxycholic acid effects. Indeed, by targeting the mTOR pathway through *cyp7a1*‐mediated cholesterol‐to‐bile acid conversion, this flavonoid has been suggested to alleviate T2D‐induced hepatic lipid accumulation [43]. MD‐fed db/db mice, quite paradoxically, showed, among diabetic animals, the highest expression of *pck1*. This discrepancy could be more apparent than real when considering that *pck1* not only is the rate‐limiting enzyme for gluconeogenesis, but also a crucial control point for cataplerosis, maintaining metabolic flux through the Krebs cycle and removing the excess in oxaloacetate. *Pck1*‐dependent suppression of this function was revealed to be lethal or lipogenic in the liver [44]. Conversely, WD‐fed db/db mice showed significant upregulation of lipogenic genes, including *srebp1c*, *fas1*, and *cd36*, which drive triglyceride synthesis and lipid uptake. The hepatic metabolome of WD‐fed db/db mice revealed an accumulation of saturated fatty acids and long‐chain phospholipids, indicative of disrupted mitochondrial function and impaired triglyceride clearance. These findings align with their severe hepatic steatosis, hypercholesterolemia, and early signs of fibrosis, consistent with metabolic derangements observed in WD studies [45, 46]. Reflecting their liver gross morphology, WD‐fed mice also showed a hepatic overaccumulation of chenodeoxycholic acid, reported in late‐stage liver disease, including NASH and HCC. A recent metabolomics study revealed chenodeoxycholic acid as being significantly increased in both tumor tissue and in serum of HCC patients and was listed as a new biomarker of human HCC (Han et al. 2019) [47]. More recently, in preclinical models of NASH, bile acid composition in the enterohepatic circulation was found to be profoundly altered, with specific depletion of secondary bile acids, which are known to exert anti‐inflammatory effects [48]. In addition, unlike physiological or therapeutic concentrations of FXR and TGR5 receptor agonists, which may elicit protective effects on mitochondria [49], excessive chenodeoxycholic acid or dysregulated bile acid metabolism has been associated with mitochondrial dysfunction [50].

MD's antioxidant properties emerged as a key factor in countering diabetes‐induced oxidative stress. Levels of lipid peroxides, protein carbonyls, and RAGEs were normalized in MD‐fed db/db mice, in the absence of any effects on antioxidant enzymes. This suggests that the diet mitigated oxidative damage by enhancing direct scavenging of ROS and protecting against cellular damage [51, 52]. Indeed, dietary enrichment of plant‐derived antioxidants, including isolariciresinol and ergothioneine, as well as essential vitamins and their derivatives, like ascorbic acid sulfate, represent potent defense mechanisms against oxidative stress, contrasting sharply with WD that exacerbated oxidative damage and contributed to mitochondrial dysfunction and fibrosis [53]. A crucial observation was the MD's ability to preserve mitochondrial quality control mechanisms, critical for cellular energy homeostasis and metabolic regulation. MD‐fed db/db mice exhibited higher mitochondrial DNA content, increased levels and activity of OXPHOS enzymes, and improved mitochondrial dynamics. The expression of mitochondrial dynamics proteins such as DRP1 and MITOFUSIN 2, along with regulators of mitophagy like PINK1 and Parkin, was maintained in MD‐fed db/db mice, suggesting enhanced mitochondrial integrity and adaptive capacity. These observations are in line with previous studies on in vivo and in vitro effects of purified MD metabolites, such as oleuropein, hydroxytyrosol, and others in the context of liver disease models [41, 42, 54, 55, 56, 57], and furnish original information on liver mitochondrial effects of an MD mix as a whole. In parallel, MD did not significantly alter hepatic levels of autophagy modulators (mTOR, AMBRA, and p62) as, at multiple levels, diabetes and WD did, stimulating, at the same time, AMPK activity (Thr172 phosphorylation), a crucial control point for liver lipid catabolism, oxidative capacity, mitophagy, and autophagy, very recently shown to be targeted by several plant metabolites eliciting hepatoprotective effects against fatty liver [58, 59]. In the case of nutrient overload, fat utilization by mitochondria is a crucial balancing mechanism. Accumulating information indicates that MD, although not significantly contrasting diabetes‐induced down‐regulation of the molecular PPARα‐CPT1 axis, one of the major upstream controllers of mitochondrial fat oxidation, was able to qualitatively/quantitatively restore the liver pool of acylated carnitine derivatives, suggesting normalized fatty acid flux toward mitochondria, β‐oxidation, as well as preserved hepatic response to hypoxia [60, 61]. Preserved homeostasis of hepatic acylated carnitine derivatives in MD‐fed db/db animals can also explain, at least in part, the metabolic tolerance of these animals to fructose, the most abundant monosaccharide of their diet [62]. Additionally, while high‐fructose diets are often associated with adverse metabolic outcomes in several animal models of metabolic disease—including the upregulation of lipogenic genes, insulin resistance, and mitochondrial dysfunction [63, 64]—it is important to note that the MD used in this study was not a purified high‐fructose formulation. Instead, it was a complex mixture derived from natural fruit and vegetable matrices, which also provided substantial amounts of dietary fiber, polyphenols, and other bioactive compounds. These components are known to counteract the detrimental effects of fructose by slowing its absorption, enhancing satiety, modulating gut microbiota composition, enhancing antioxidant defenses, and reducing hepatic inflammation [65, 66], here likely eliciting a matrix and co‐delivery effect. In contrast, WD‐fed db/db mice displayed impaired mitochondrial quality control, characterized by increased mitochondrial fission and reduced autophagic clearance. This likely contributed to their profound metabolic dysfunctions, as disruptions in mitochondrial dynamics and autophagy have already been linked to insulin resistance, NAFLD, and T2D progression [67, 68, 69, 70]. MD also preserved diabetes‐induced disorders in hepatic creatine metabolism, an important determinant of energy states, crucially impacting the phosphorylcreatine/creatine ratio, mitochondrial function, lipid metabolism, and amino acid pools [71]. Because of the link between liver and skeletal muscle creatine metabolism, the elevated hepatic creatine levels observed in WD‐fed db/db mice might be a consequence of the observed muscle mass loss. Of note, WD‐fed db/db mice exhibited signs of fibrosis, associated with oxidative damage, lipid peroxidation, hypercholesterolemia, and mitochondrial dysfunction. Conversely, fibrosis was absent in MD‐fed db/db mice despite their hyperglycemic state. This suggests that MD's beneficial effects on lipid metabolism, oxidative stress reduction, and mitochondrial function collectively protect against fibrotic progression. These findings align with previous studies demonstrating the anti‐fibrotic properties of MD components, such as polyphenols and omega‐3 PUFAs, these last ones having been shown to reduce hepatic fibrosis by modulating inflammatory and fibrotic pathways [72, 73]. At the same time, they pave the way for future studies on emerging molecular targets relevant to liver health, such as proprotein convertase subtilisin/kexin type 9 (PCSK9), a regulator of cholesterol metabolism, mitochondrial function, and inflammation [74], and now recognized as being modulated by polyphenols, omega‐3 PUFA, and plant sterols [75]. In this context, a recent cross‐sectional study reported a positive association between higher intake of dietary polyphenols and comprehensive healthy aging scores [76]. Since liver function is integral to systemic homeostasis and metabolic resilience during aging, these findings support the notion that polyphenol‐rich dietary patterns like the MD may sustain liver health by attenuating inflammation and oxidative stress, thereby contributing to broader healthy aging trajectories.

## 5 Conclusions

The functional role of the MD cocktail reported in the present study in supporting liver health encompasses multiple aspects, including antioxidant activity and the regulation of metabolic pathways. The effects involve preserving redox balance, reducing oxidative damage, lowering fat accumulation, and maintaining mitochondrial quality control. Reflecting the holistic nature of the MD, the formulated MD‐based food mix likely delivered specific components exerting distinct effects: polyphenols may have conferred antioxidant protection and improved mitochondrial efficiency; EVOO‐ and nuts‐derived MUFA may have supported membrane integrity and lipid metabolism; fish‐ and nuts‐derived PUFA may have elicited ant‐inflammatory and antifibrotic effects; legume‐ and whole cereal‐derived fibers could have beneficially influenced gut‐liver axis signaling. This study reinforces MD as a beneficial dietary model for managing diabetes and obesity, particularly in mitigating liver dysfunction and mitochondrial impairments through the control of lipid and bile acid metabolism, and possibly by modulating the exchange of beneficial metabolites along the gut‐liver axis [77]. Exploring gut microbiota alterations, delineating microbiota‐host interactions, and assessing the durability of MD‐mediated benefits over time appear of specific relevance, as they may offer additional mechanistic insight into the observed metabolic and hepatic improvements. While further studies are needed to confirm its long‐term impact and clinical applicability, MD offers a promising dietary strategy to address the growing global burden of MetS and associated complications and promotes the identification of specific bioactive compounds for targeted dietary interventions or nutraceuticals.

## Conflicts of Interest

The authors declare no conflicts of interest.

## Supporting information




**Supporting file 1**: mnfr70210‐sup‐0001‐SuppMat.pdf

**Supporting file 2**: mnfr70210‐sup‐0002‐TableS2.xlsx

## Data Availability

The datasets generated and/or analyzed during the current study are available from the corresponding author upon reasonable request.
