# Supplementary material for: A Mediterranean Diet‐Based Food Mix Ameliorates Diabetes‐ and Obesity‐Associated Liver Alterations Through Mitochondrial and Metabolic Reprogramming
Source: Mol Nutr Food Res. 2025 Sep 1;69(21):e70210. doi: 10.1002/mnfr.70210 (PMC12581731; doi:10.1002/mnfr.70210)
Supplement: Supplementary file 1 — Supporting file 1: mnfr70210‐sup‐0001‐SuppMat.pdf [file MNFR-69-e70210-s001.pdf]

SUPPLEMENTARY INFORMATION

Materials and Methods

Fatty acid methyl esters (FAMEs) method

To determine the fat content of the three diets, a modified FAMEs method was applied (Anuar et al., 2012). For lipid extraction, 1 g of dried powdered sample was treated with 5 mL of hexane, three times, to dissolve lipids. The mixture was shaken for 30 s, and the supernatant was collected. Magnesium sulfate was added, and the sample was centrifuged (at 5000 rpm for 5 min). The hexane layer was then recovered and dried under nitrogen flow. Triplicate extractions were performed for each diet. For transesterification of lipids, 1 mL of 1% sulfuric acid in methanol was added to each sample, vortexed for 30 s, and incubated at 50 °C for 2 h. After cooling, 5 mL of 7% w/v NaCl solution was added, followed by three rounds of hexane extraction. The hexane layer was washed with 1 mL of 2% w/v sodium bicarbonate, dried with magnesium sulfate, and centrifuged (at 5000 rpm for 5 min). The hexane extract was transferred to a volumetric flask, with dichloronane (DCN) added as an internal standard. For GC-MS analysis, derivatized samples were analyzed using an Agilent-7820 GC System with an Agilent 5977E mass spectrometric detector. A DB-23 capillary column (30 m length, 0.25 mm diameter, 0.25 µm film) was used. Sample injection (1 µL) was in split mode (50:1). Helium served as the carrier gas at 20 mL/min. The oven temperature was initially 50 °C, held for 1 min, with an initial ramp rate of 25 °C/min until 200 °C, held for 8 min, with a second ramp rate of 5 °C/min until 230 °C, held for 6 min. Mass spectra were recorded in the *m/z* range 40–250, with DCN serving as the internal standard for quantification. Data were processed using MassHunter Workstation Software.

Crude protein content by the Kjeldahl method

Protein content of the diet samples was determined following a modified Kjeldahl method (Rizvi et al., 2022). Each diet sample (0.7 g) was digested with 30 mL sulfuric acid in the presence of two

kjtabs VCT (5 g potassium sulfate, 0.15 g copper sulfate, 0.15 g titanium dioxide) and 3 drops of octanol. The digestion was performed in a DK6 heating block at 300 °C for 40 min, followed by 90 min at 420 °C. The samples were then cooled to room temperature. The digested samples were steam distilled with 50 mL of water and 60 mL of 40% w/v NaOH into 30 mL boric acid containing a 3% w/v indicator. A 90-second pause followed each addition, and the steam distillation was carried out for 4 min at full steam power (100%). Distilled samples were titrated against 0.2 N sulfuric acid. The protein content was calculated based on the nitrogen percentage using the following equation:

$$\% \, protein = \frac{(Sample\ titre - Blank\ titre)\ x\ N\ of\ acid\ x\ 1.4007}{Sample\ mass\ in\ grams}\ x\ 6.25$$

where 6.25 is a correction factor for grains, and N represents the normality of the acid.

Metabolite Analysis

Metabolite analysis of the diet samples was carried out following a modified method from Ledbetter et al. (2022). Defatted diet samples (100 mg) were mixed with 3 mL of 60:40 methanol solution, vortexed for 30 min at 1000 rpm, and centrifuged at 6000 rpm. A 250 µL aliquot of the supernatant was transferred to an Eppendorf tube containing 100 µL of cycloleucine (0.1 mg/mL) as an internal standard. The sample was evaporated to dryness at 30 °C for 4 h. The dried sample was incubated with 150 µL methoxyamine (20 mg/mL in pyridine) at 60 °C for 4 h. Then, 150 µL of N-methyl-N-trimethylsilyl-trifluoroacetamide (MSTFA) was added, followed by incubation at 45 °C for 45 min. The final sample was transferred to a vial for GC-MS analysis. Analysis was performed using an Agilent-7820 GC System with a 5977E mass spectrometric detector. A ZB-5MS column (30 m length, 0.25 mm diameter, 0.25 µm film) was used. Samples (1 µL) were injected in pulsed splitless mode, with helium as the carrier gas at 20 mL/min. The oven temperature started at 70 °C for 2 min, then increased at 5 °C/min to 260 °C, followed by 15 °C/min to 290 °C, held for 5 min. Mass spectra were recorded in the *m/z* range 50–500. Cycloleucine quantification was conducted in

selected ion monitoring (SIM) mode using *m/z* 156.1 with a dwell time of 200 ms. Peak areas of metabolites were compared to cycloleucine, and data were analyzed using MassHunter Workstation Software.

Polyphenol Extraction and Total Phenolic Content (TPC) Assay

Polyphenols were extracted from diet samples following a modified method from Alonso-Salces et al. (2004). Dried diet samples were dissolved in 60% v/v methanol in water at a concentration of 10 mg/mL. The mixture was vortexed for 2 min and extracted using a Thermomixer Comfort (Eppendorf) at 500 rpm for 30 min, at room temperature. The solution was then centrifuged at 5000 rpm for 15 min, and the supernatant was collected. The total phenolic content (TPC) assay was performed according to de Falco et al. (2018). For each sample, 27 μL of polyphenol extract was mixed with 432 μL of 7.5% w/v sodium carbonate and 540 μL of Folin-Ciocalteu reagent (diluted 1/10 in water). The mixture was incubated at 50 °C for 5 min, and the absorbance was measured at 760 nm. Each sample was analyzed in triplicate. TPC was expressed in gallic acid equivalents (μg GAE/g), and a calibration curve (0–3 mM) was used to quantify the TPC in the diet samples.

ABTS radical scavenging activity assay

The ABTS assay was performed as described by de Falco et al. (2018), using the reduction of 2,2'-azinobis-(3-ethylbenzothiazoline-6-sulfonic acid) (ABTS) radical cation. ABTS radicals were generated by mixing 4.912 mL of 7 mM ABTS in water with 88 μL of 140 mM potassium persulfate, stored at 4 °C in the dark, for 24 h. The ABTS stock solution was diluted 1:90 v/v in water to achieve an optical density of 0.7–0.8 at 734 nm. To measure antioxidant activity, 1.0 mL of ABTS working solution was mixed with 100 μL of the sample, and absorbance was measured after 2.5 min. Blank and control samples used water, and all measurements were done in triplicate. Inhibition percentage values were calculated using the formula:

$$\% \text{ Inhibition} = (1 - (AU\text{sample}/AU \text{ ABTS working solution})) \times 100.$$

Results were expressed in mmol Trolox equivalent antioxidant capacity (TEAC/g) based on a calibration curve with Trolox (15–250 μM).

DPPH radical scavenging activity assay

The antiradical activity of the diet samples was assessed using the 2,2-diphenyl-1-picrylhydrazyl (DPPH) method, based on Blois (1958) with slight modifications. A 1 mM DPPH stock solution in methanol was diluted 1:13 to obtain a working solution with an absorbance of 0.800–0.900 at 517 nm. For analysis, 1.0 mL of DPPH working solution was mixed with 20 μL of the sample, incubated for 10 min, and the resulting absorbance was measured. All samples were analyzed in triplicate. Inhibition percentage values were calculated using the formula:

$$\% \text{ Inhibition} = (1 - (AU\text{sample}/AU \text{ DPPH working solution})) \times 100.$$

Results were expressed as gallic acid equivalents (GAE/g) using a calibration curve with gallic acid (0–150 mM).

Ferric reducing ability power (FRAP) assay

The antioxidant power of the diet samples was determined by ferric reducing ability based on the reduction of Fe(III) to Fe(II) and the formation of a ferrous-TPTZ complex, measurable at 593 nm (Benzie and Strain, 1996). The FRAP solution consisted of 25 mL of 300 mM sodium acetate (pH 3.6), 2.5 mL of 10 mM TPTZ (in 40 mM HCl), and 2.5 mL of 20 mM FeCl3•6H2O. For analysis, 900 μL of FRAP solution was added to 100 μL of the sample and incubated for 4 min, at room temperature. Absorbance was measured at 593 nm. All measurements were done in triplicate. Results were calculated using two standard curves: one with Fe(II) (50–500 μM) to quantify Fe(III) reduction and another with gallic acid (15–120 μM) for antioxidant capacity, expressed as μg gallic acid equivalents (GAE/g).

Metabolomic analysis of liver tissues

For metabolomic analysis of liver samples, we referred to Gegner et al, 2022 for the extraction protocol and to De Filippo et al 2024 for the mass spectrometry layout. In both cases, we introduced some modifications. Briefly, 60 mg of liver sample were mixed with 120 mg of glass beads and 540 µL of cold methanol. Methanolic suspensions were homogenized in a Tissuelyser (Retsch, Haan, Germany) for 30 min, at 30 Hz, for 6 times. Homogenized samples were placed in an ultrasonic bath on ice for 5 min, incubated for 5 min at 37 °C, and resuspended in 600 µL of water. After centrifugation of samples at 16,000 rpm for 10 min, the supernatants were transferred to new tubes and dried in the Speed Vac Concentrator (Thermo Fisher Scientific, USA), overnight (without temperature). Finally, the samples were reconstituted in 180 µL of a mixture of isopropanol:water 85:15 v/v, placed in an ultrasonic bath on ice for 1 min and centrifuged at 16,000 rpm for 10 min, before transferring to autosampler vials.

Liquid chromatography tandem high resolution mass spectrometry (LC-MS/MS)

An Exploris 120 quadrupole Orbitrap high-resolution mass spectrometer and a Vanquish Core liquid chromatographic system (Thermo Fisher Scientific, Bremen, Germany) were used for metabolites screening, annotation and identification. Compounds were separated at 40 °C through a C18 core shell column (Kinetex PS, 100 x 2.1 mm, 2.6 µm, Phenomenex, Torrance, CA) with the following gradient of solvent B (minutes/%B): (0/15), (0.5/15), (7/99), (12/99). The flow rate was 0.2 mL/min, and the mobile phases consisted of 0.1% formic acid in water (solvent A) and 0.1% formic acid in acetonitrile (solvent B). Liver homogenate sample runs were acquired in polarity switching mode in the *m/z* range 70-800. Heated electrospray interface (H-ESI) static spray voltage was -3.2 kV for negative ions and 3.5 kV for positive ions. Ion transfer tube and vaporizer temperature were set at 300 and 280 °C, while sheath gas flow and auxiliary gas flow were 45 and 10 arbitrary units, respectively. The analyzer resolution was set at 60,000 (FWHM at *m/z* 200) and profile data were acquired with a normalized automatic-gain control (AGC) target of 100%. Compounds identification was achieved through a sequence batch based on paired identification runs by

injecting quality controls spiked with analytical standard mix and scanning the ions through differential ranges working exclusively in positive or negative data dependent scanning mode (ddMS2). Upon procedural blank spectra subtraction, three intertwined methods with the same chromatographic elution profile and differential scan ranges (*m/z* 70-250, *m/z* 240-550, *m/z* 540-800, resolution 60,000 FWHM at *m/z* 200) were implemented to add a third dimension to the isotopic patterns and molecular formulas. For ddMS2 top 4 ion peak experiments, an isolation window of *m/z* 1.2 was used, while normalized collision energy was fixed at 20, 55 and 70%. Data profiles were acquired with an intensity threshold fixed at 50,000 (area counts); the dynamic exclusion was customized by considering as time window 3.0 s and a mass tolerance of 5 ppm. Signal correction was obtained through scan-to-scan internal calibrant spiking with fluoranthene in positive ion mode ($[M]^+$ *m/z* 202.0777) and negative ion mode ($[M]^-$ *m/z* 202.0788) in both full scan and ddMS2 mode (EASY-IC, Thermo Fisher Scientific). Data were collected using Xcalibur 4.5 and Free Style software (Thermo Fisher Scientific, Waltham, MA).

Untargeted metabolomics and targeted metabolites quantification

Raw files were loaded in Compound Discoverer 3.3 (Thermo Fisher Scientific, San José, CA). An untargeted metabolomic workflow based on metabolites identification was used to find and characterize differences between db/m and db/db mice, ages at the sacrifice and different diets (MD, SD and WD). The procedure involved the retention time alignment and detection of known and unknown compounds for grouping of samples. Upon definition of the elemental composition, exact mass values, chemical formulas and fragmentation spectra, compounds were matched with information reported in publicly available databases, such as mzCloud (*www.mzcloud.org*), ChemSpider (*www.chemspider.com*), human metabolome database (*www.hmdb.ca*) and KEGG (*www.genome.jp/kegg/compound*) and with analytical standards. A supplementary search was performed in phenol-explorer (*www.phenol-explorer.eu*) and through an internal mass list generated starting with the flavonoid structure database to investigate the presence of phytochemicals

metabolites arising from the Mediterranean diet. We reported for each compound an identification level ranging from 1 (compound identification upon matching to analytical standards) to 2 (compounds annotation based on mass spectra, isotopic pattern, chemical formula, mass accuracy and matching with databases) according to Metabolomics standard initiative (MSI) level (Sumner et al., 2007) (Supporting Information Table S2). After correction for quality control samples, post-processing nodes performed descriptive statistics and differential analysis working on two technical replicates arising from one bio-replicates representative of two observations for each sample. Hypothesis test was performed by one-way ANOVA model with Tukey as *post-hoc* test. For volcano plots and ratio among sample groups, p-values were adjusted by Benjamini-Hochberg algorithm. Principal component analysis (PCA) and log fold changes were obtained in Compound Discoverer environment. Quantification of target compounds was performed through the standard addition technique according to the standard availability and chemical similarity (Supporting Information Table S3).

Reverse Transcription (RT) quantitative (q) PCR

RNA was isolated from liver using the TRIZOL standard protocol (Invitrogen, California, USA). RNA (1 μg) was used to generate cDNA strands in a 20-μl-reaction volume (Quanti Tect Reverse Transcription Kit, Qiagen, Hilden, Germany). qPCR was carried out with 50 nM gene-specific primers and SYBR Green supermix (Applied Biosystems, Foster City, California, USA) using standard cycle parameters on a QuantStudio 5 System (Thermo Fisher Scientific, Waltham, Massachusette, USA). The transcript abundance was calculated by the 2-ΔΔCT method and normalized to the expression of the housekeeping gene, glyceraldehyde 3-phosphate dehydrogenase (GAPDH). PCR primers were designed using the Primer 3 program (0.4.0) and synthesized by Eurofins Genomics (Giacco et al., 2022). Primer sequences are reported in Supporting Information Table S1A.

Separation of respiratory complexes by BN-PAGE and histochemical staining for in-gel activity

Liver fragments were homogenized in ice-cold isolation buffer (220 mM mannitol, 70 mM sucrose, 20 mM Tris-HCl, 1 mM EDTA, 5 mM EGTA, pH 7.4) using a Potter-Elvehjem homogenizer. The homogenate was centrifuged at 500 x *g* for 10 min, and then at 3000 x *g* for 10 min to isolate the mitochondrial pellet. Mitochondrial membranes were solubilized with 10% w/v dodecyl-maltoside and separated by BN-PAGE on 6-13% gradient gels (Scagger et al., 1995). Enzymatic activity staining for complexes I, II, and IV was performed using NADH/NTB, sodium succinate/NTB, and cytochrome c/DAB reactions, respectively (Zerbetto et al., 1997). Gels were fixed in methanol/acetic acid and stained with Coomassie Blue for total protein visualization. Each BN-PAGE analysis was repeated independently at least three times. For each run, parallel Coomassie staining of total protein was used as loading controls. Densitometric analysis was conducted using a GS-800 densitometer and QuantityOne software. (Bio-Rad). Scanned gel-images were processed for the removal of background and automatic detection of bands. The areas of the bands were expressed as absolute values (arbitrary units).

Determination of the relative DNA mitochondrial copy number

Genomic DNA was extracted and purified from approximately 20 mg of frozen liver using QIAGEN Genomic-tip 20/G and Genomic DNA Buffer Set (Qiagen, Venlo, the Netherlands). Purity and quantity of extracted DNA was determined spectrophotometrically at 260 and 280 nm. The mitochondrial DNA (mtDNA) content was measured by real-time PCR using a QuantStudio 5 System (Thermo Fisher Scientific, Waltham, Massachusetts, USA) essentially as reported in Giacco et al. (2022). The amplification of mitochondrial cytochrome c oxidase subunit II (COII, mitochondrial-encoded gene), cytochrome b (Cytb, mitochondrial-encoded gene) and β-actin (nuclear-encoded gene) were examined. The primer sequences used were as follows: COII: 5’-TGAGCCATCCCTTCACTAGG-3’(sense)/5’-TGAGCCGCAAATTTCAGAG-3’(anti-sense); Cytb: 5’-TACCTGCCCCATCCAACATT-3’(sense)/5’-TAAGCCTCGTCCGACATGAA-3’(anti-

sense); β-actin: 5’CTGCTCTTTCCCAGATGAGG-3’(sense)/5’-CCACAGCACTGTAGGGGTTT-3’(anti-sense). Reactions were carried out in the presence of 1x iTaq Universal SYBR Green Supermix (BioRAD), 0.5 μM of each forward and reverse primer, and 50 ng genomic DNA. The threshold cycle number (Ct) was calculated using Design and Analysis software v1 5.1 (Applied Biosystems, Thermo Fisher Scientific, US) and an automated setting of the baseline. The relative mtDNA copy number (Rc) was determined as described in Rooney et al. (2015), using the equations:

$Rc = 2 \times (2^{\Delta Ct})$

ΔCt = average Ct nuclear DNA − average Ct mtDNA.

References

S.T. Anuar, S. M. Mugoc, J. M. Curtisa, *Analytical Methods.* **2015**, 7, 5898.

N. B. Rizvi, S. Aleem, M. R. Khan, S. Ashraf, R. Busquets, *Molecules*. **2022**, 27, 814.

M. Ledbetter, S. Blidi, S. Ackon, F. Bruno, K. Sturrock, N. Pellegrini, A. Fiore, *Heliyon*. **2021**, 7, e07441.

R.M. Alonso-Salces, A. Barranco, E. Corta, L.A. Berrueta, B. Gallo, F. A. Vicente, *Talanta*. **2005**, 65, 654.

B. De Falco, A. Fiore, R. Bochicchio, M. Amato, V. Lanzotti, *Industrial Crops and Products*. **2017**, 112, 584.

M.S. Blois, *Nature*. **1958**, 181, 1199.

I.F. Benzie, J.J. Strain, *Anal. Biochem*. **1996**, 239, 70.

H.M. Gegner, N. Mechtel, E. Heidenreich, A. Wirth, F.G. Cortizo, K. Bennewitz, T. Fleming, C. Andresen, M. Freichel, A.A. Teleman, J. Kroll, R. Hell, G. Poschet, *Front. Chem*. **2022**, 10, 869732.

C. De Filippo, S. Chioccioli, N. Meriggi, A.D. Troise, F. Vitali, M.M. Monroy, S. Özsezen, K. Tortora, A. Balvay, C. Maudet, N. Naud, E. Fouché, C. Buisson, J. Dupuy, V. Bézirard, S. Chevolleau, V. Tondereau, V. Theodorou, C. Maslo, P. Aubry, C. Etienne, L. Giovannelli, V. Longo, A. Scaloni, D. Cavalieri, J. Bouwman, F. Pierre, P. Gérard, F. Guéraud, G. Caderni, *Microbiome*. **2024**, 12, 180.

L.W. Sumner, A. Amberg, D. Barrett, M.H. Beale, R. Beger, C.A. Daykin, T.W. Fan, O. Fiehn, R. Goodacre, J.L. Griffin, T. Hankemeier, N. Hardy, J. Harnly, R. Higashi, J. Kopka, A.N. Lane, J.C. Lindon, P. Marriott, A.W. Nicholls, M.D. Reily, J.J. Thaden, M.R. Viant, *Metabolomics*. **2007**, 3, 211-221.

A. Giacco, T. Peluso, F. Cioffi, S. Iervolino, G. Mercurio, L. Roberto, C. Reale, M. Colella, M. De Felice, M. Moreno, C. Ambrosino, E. Silvestri, *J. Endocrinol*. **2022**, 253, 115.

H. Schagger, W.A. Cramer, G. Jagow, *Anal. Biochem*. **1994**, 217, 220.

E. Zerbetto, L. Vergani, F. Dabbeni-Sala, *Electrophoresis*. **1997**, 18, 2059.

Supporting Information Table 1. (A) List of primers used for RTqPCR analysis. (B) List of antibodies used for Western blot analysis, molecular mass of the target protein and used experimental conditions.

(A)

**Gene**	**Primer sequence (5'-3')**	
	**Forward**	**Reverse**
Abcg5	AATTTTGGGGGAATTTCCAG	GTCCTGTGGTTGGCTCATCT
Acc1	TTCACTGTGGCTTCTCCAGC	GACCACCGACGGATAGATCG
Chrebp1	GCATCCTCATCCGACCTTTA	GATGCTTGTGGAAGTGCTGA
Cd36	ATGGGCTGTGATCGGAACTG	AGCCAGGACTGCACCAATAAC
Cpt1	TCTGGATGGCTATGGTCAAG	GGCCTCACAGACTCCAGGTA
Cyp7a1	CTCCGGGCCTTCCTAAATCA	ACAGCGTTAGATATCCGGCT
Dgat1	GGTTCCGTGTTTGCTCTGGCAT	CCACTGACCTTCTTCCCTGTAG
Dgat2	CTGTGCTCTACTTCACCTGGCT	CTGGATGGGAAAGTAGTCTCGG
Fas1	GACTCGGCTACTGACACGAC	CGAGTTGAGCTGGGTTAGGG
G6P	CTCACTTTCCCCACCAGGTC	GAATCCAAGCGCGAAACCA
Gapdh	AGGTCGGTGTGAACGGATTTG	TGTAGACCATGTAGTTGAGGTTCA
Hmgcr	CAAGGAGCATGCAAAGACAA	GCCATCACAGTGCCACATAC
Ldlr	GACCCAGAGCCATCGTAGTG	AACCCAATAGAGACGGCCAC
Lpl	GAGACTCAGAAAAAGGTCATC	GTCTTCAAAGAACTCAGATGC
Pck1	AGGCAGTGAGGAAGTTCGTG	AGCCAGCCAACAGTTGTCAT
Pgc1α	GTCAACAGCAAAAGCCACAA	GTGTGAGGAGGGTCATCGTT
Pparα	ACGTTTGTGGCTGGTCAAGT	GCTCTCTGTGTCCACCATGT
Pparγ	AGGCCGAGAAGGAGAAGCTGTTG	TGGCCACCTCTTTGCTCTGCTC
Srebp1c	CGGCTCTGGAACAGACACT	CTGTCTCACCCCCAGCATAG
Srebp2	ACTGACCAGCACCCATACTC	CAGGAGGAGAGTTGGAACCA

(B)

Antibody	Cat. number	Dilution	Validation
AMPK (62 kDa)	Cell Signaling #2532	1:1000 in 1XTBST with 5% BSA	*
AMBRA (140 kDa)	Cell Signaling #24907	1:1000 in 1XTBST with 5% non fat dry milk	*
β ACTIN (42 kDa)	Gene Tex GTX109639	1:3000 in 1XTBST with 5% non fat dry milk	**
CATALASE (60 kDa)	Sigma-Aldrich 0979	1:2000 in 1XTBST with 5% non fat dry milk	****
COLLAGEN IV ALFA 1 (160 kDa)	Abcam ab227616	1:500 in 0.5XPBST with 5% BSA	N.A.
DRP1 (85 kDa)	Abcam ab56788	1:500 in 1XTBST with 5% non fat dry milk	N.A.
GAPDH (37 kDa)	Abcam ab9484	1:1000 in 1XTBST with 5% BSA	N.A.
GPX4 (22 kDa)	Abcam ab125066	1:5000 in 1XTBST with 5% non fat dry milk	**
LC3B (14-16 kDa)	Abcam 192890	1:1000 in 1XTBST with 5% non fat dry milk	**
MITOFUSIN2 (80 kDa)	Abcam ab56889	1:1000 in 1XTBST with 5% non fat dry milk	N.A.
MT-MMP-1 (C-7) (63 kDa)	Santa Cruz Biot. Sc-377097	1:1000 in 0.5XPBST with 5% non fat dry milk	***
mTOR (289 kDa)	Cell Signaling #2983	1:1000 in 1XTBST with 5% BSA	*
OXPHOS cocktail	Abcam Ab110413	1:250 in 1XTBST with 10% non fat dry milk	N.A.
P62 (62 kDa)	Cell Signaling #5114	1:1000 in 1XTBST with 5% BSA	*
PARKIN (50 kDa)	Cell Signaling #4211	1:1000 in 1XTBST with 5% non fat dry milk	*
PEROXIREDOXIN 3 (26 kDa)	Abcam ab73349	1:1000 in 1XTBST with 5% non fat dry milk	N.A.
Phospho-AMPK (Ser473) (62 kDa)	Cell Signaling #2535	1:1000 in 1XTBST with 5% BSA	*
Phospho-mTOR(Ser2448) (289 kDa)	Cell Signaling #5536	1:1000 in 1XTBST with 5% BSA	*
PINK1 (60 kDa)	Abcam ab186303	1:1000 in XTBST with 5% BSA	N.A.
PGC1α (110 kDa)	Sigma-Aldrich Ab3242	1:1000 in 1XTBST with 5% non fat dry milk	***
RAGE (A-9) (46 kDa)	Santa Cruz Biot. sc-365154	1:500 in 0.5XPBST with 5% non fat dry milk	***
SOD2/MnSOD (27 kDa)	Abcam ab613533	1:5000 in 1XTBST with 5% non fat dry milk	N.A.
TIMP-2 (3A4): sc-21735 (23 kDa)	Santa Cruz Biot. Sc-21735	1:1000 in 0.5XPBST with 5% non fat dry milk	***
TFAM (28 kDa)	Abcam ab131607	1:1000 in 1XTBST with 5% non fat dry milk	N.A.
VEGF (21 kDa)	Santa Cruz Biot. sc-57496	1:1000 in 0.5TPBST with 5% non fat dry milk	***
VDAC (30 kDa)	Abcam ab34726	1:1000 in 1XTBST with 5% non fat dry milk	N.A.

* tested on endogenous high and low expressing cell lines
** tested through knockout/ knockdown controls
*** tested through peptide competition assays
****tested on relevant cancer cells

Supporting Information Table S3. Identified and annotated compounds that were also quantified in liver tissues of db/m and db/db mice using reference analytical standards. For all the compounds listed, an identification level of 1 was achieved by considering isotope pattern distribution, fragmentation spectra, and matching to analytical standards that are part of the same molecular class. Spectra were manually annotated and checked with publicly available databases as described in the experimental section of the Supporting Information.

Name	Formula	Annotated MW	Calc. MW	m/z	RT [min]
tau-Methylhistidine	C7 H11 N3 O2	169.08513	169.08517	170.09245	0.897
Trimethylamine N-oxide	C3 H9 N O	75.06841	75.06845	76.07572	1.030
D-(+)-Maltose	C12 H22 O11	342.11621	342.11583	387.11407	1.038
Acetyl-L-carnitine	C9 H17 N O4	203.11576	203.11573	204.12301	1.041
Creatine	C4 H9 N3 O2	131.06948	131.06945	132.07672	1.042
Dimethylglycine	C4 H9 N O2	103.06333	103.06324	104.07051	1.047
Propionyl-carnitine	C10 H19 N O4	217.13141	217.13137	218.13864	1.072
Anthranilic acid	C7 H7 N O2	137.04768	137.04769	138.05497	1.073
L-Ergothioneine	C9 H15 N3 O2 S	229.0885	229.08843	230.09571	1.084
DL-Tryptophan	C11 H12 N2 O2	204.08988	204.08982	205.09714	1.235
D-(+)-Glucose	C6 H12 O6	180.06339	180.0634	179.05612	1.538
2-Coumaric acid	C9 H8 O3	164.04734	164.04734	163.04007	1.813
N-Acetylkynurenine	C12 H14 N2 O4	250.09536	250.09545	251.10273	3.516
Isolariciresinol 9-O-beta-D-glucoside	C26 H34 O11	522.21011	522.21001	521.20273	4.747
Corticosterone	C21 H30 O4	346.21441	346.21449	347.22177	5.052
Dodecanoyl-carnitine	C19 H37 N O4	343.27226	343.27231	344.27958	5.536
Heptadecanoyl-carnitine	C24 H47 N O4	413.35051	413.35052	414.3578	6.675
PS(20:0/0:0)	C26 H52 N O9 P	553.33797	553.33795	552.33067	7.112
Chenodeoxycholic acid	C24 H40 O4	392.29266	392.29235	437.29057	7.160
PS(21:0/0:0)	C27 H54 N O9 P	567.35362	567.35332	566.34604	7.460
PS(12:0/15:1[9Z])	C33 H62 N O10 P	663.41113	663.41057	664.41794	8.272
Myristic acid	C14 H28 O2	228.20893	228.20886	227.20159	8.797
PS(12:0/18:3[6Z,9Z,12Z])	C36 H64 N O10 P	701.42678	701.42683	700.41948	8.931
Linoleic acid	C18 H32 O2	280.24023	280.24022	279.23295	9.119
Fructoselysine	C12 H24 N2 O7	308.15835	308.15841	309.16568	0.889
L-Ascorbic acid 2-sulfate	C6 H8 O9 S	255.9889	255.98898	254.9817	1.509
Lenticin	C14 H18 N2 O2	246.13683	246.13684	247.14412	1.898
Deoxycholic acid	C24 H40 O4	392.29266	392.29223	391.28482	7.272
3-hydroxyanthranilic acid	C7 H7 N O3	153.04259	153.0428	154.04988	1.230
L-Kynurenine	C10 H12 N2 O3	208.08479	208.08479	209.09207	1.232
2-Methylbutyroyl-carnitine	C12 H23 N O4	245.16271	245.16273	246.17001	1.235
Hexanoylcarnitine	C13 H25 N O4	259.17836	259.1785	260.18578	2.174
N-Acetyl-DL-tryptophan	C13 H14 N2 O3	246.10044	246.10048	245.09321	4.189
Phenylacetylglycine	C10 H11 N O3	193.07389	193.07391	192.06665	3.356
Phenol sulphate	C6 H6 O4 S	173.99868	173.99872	172.99144	4.403

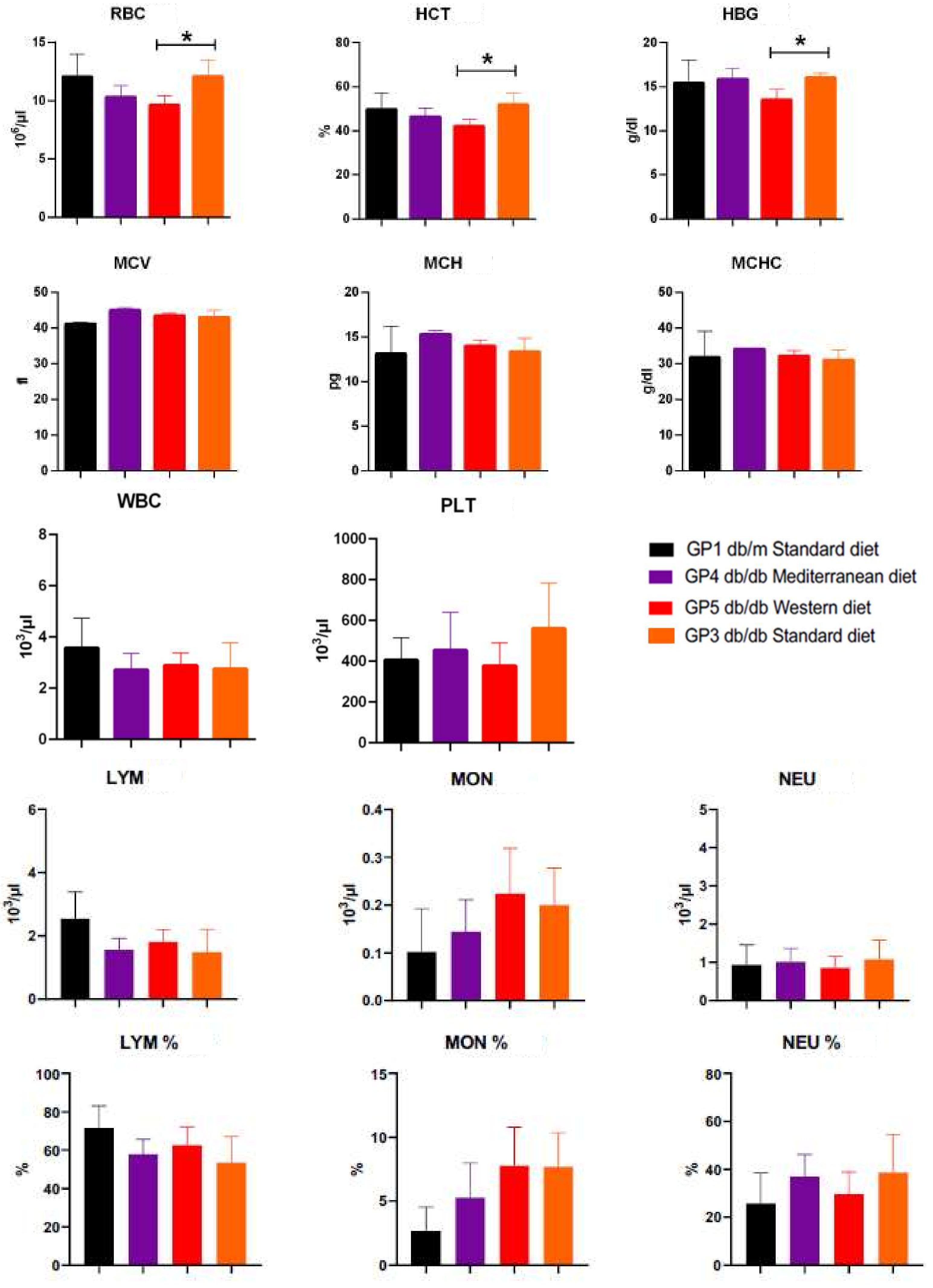
RBC
HCT
HBG
MCV
MCH
MCHC
WBC
PLT
LYM
MON
NEU
LYM %
MON %
NEU %
GP1 db/m Standard diet
GP4 db/db Mediterranean diet
GP5 db/db Western diet
GP3 db/db Standard diet
*

Supporting Information Figure S1. Effects of tested diets on blood parameters, and white blood cell and platelet counts in db/m and db/db mice. Graphic representation of the measured blood parameters including count of red blood cells (RBC), hematocrit (HCT), hemoglobin concentration (HGB), mean corpuscular volume (MCV), mean corpuscular hemoglobin (MCH) and mean corpuscular hemoglobin concentration (MCHC). Count of the different subtypes of white blood cells (WBC), platelets (PLT), lymphocytes (LYM), monocytes (MON) and neutrophils (NEU), and their respective percentage values. Shown are the mean values ± SD. n=5. One-way ANOVA, p value * <0.05.

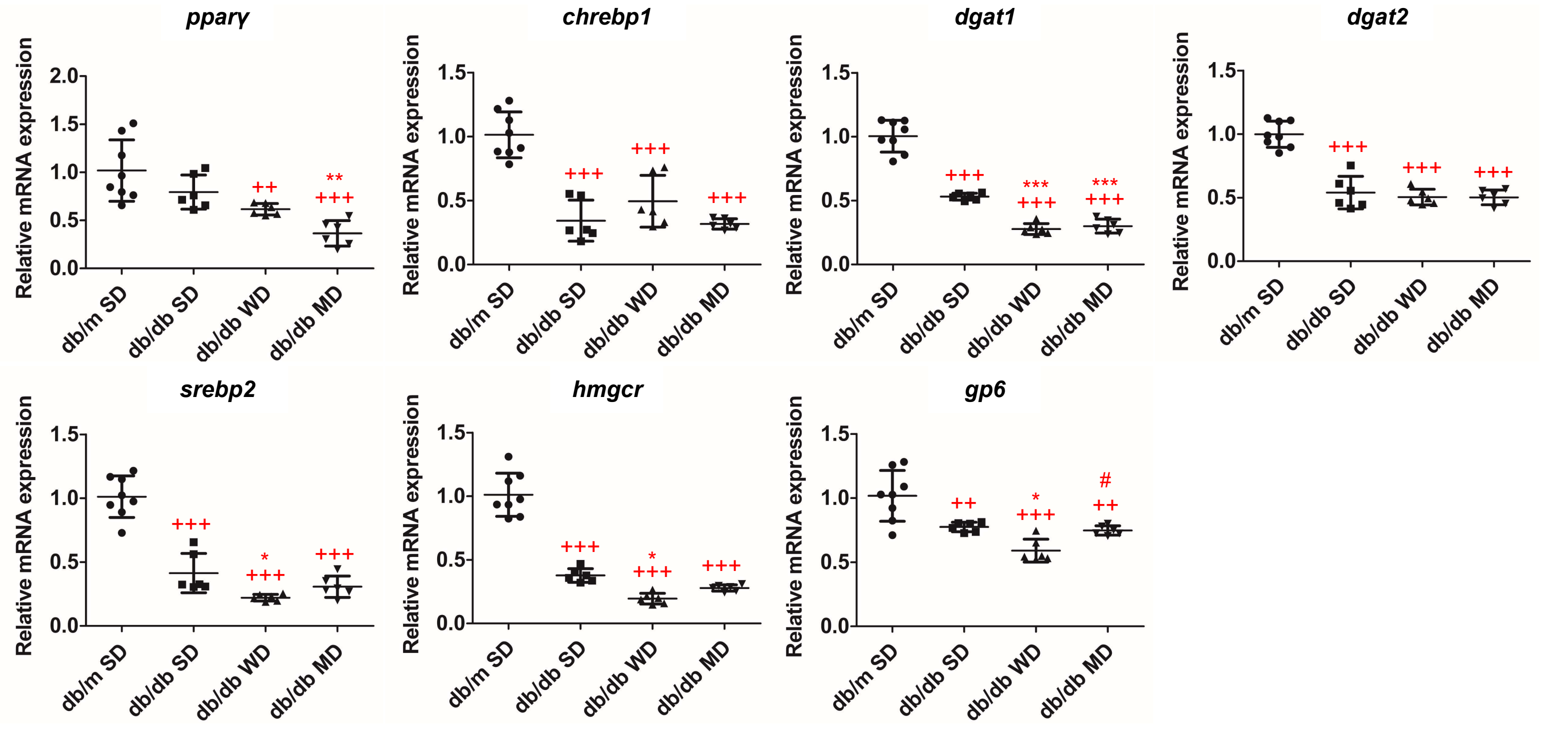

Supporting Information Figure S2. Effects of tested diets on hepatic lipid and glucose metabolism in db/m and db/db mice. mRNA expression levels of genes involved in lipogenesis (*pparγ* and *chrebp1*), triacylglycerol synthesis (*dgat1* and *dgat2*), cholesterol biosynthesis (*srebp2* and *hmgcr*) and glycogenolysis (*gp6*). Data were normalized to the values obtained for SD-fed db/m mice (control) (set as 1); shown are the mean values ± SD. n=6/8. One-way ANOVA, ++ $p < 0.01$, +++ $p < 0.001$ vs. SD-fed db/m mice; * $p < 0.05$, ** $p < 0.01$, *** $p < 0.001$ vs. SD-fed db/db mice; # $p < 0.05$ vs. WD-fed db/db mice.

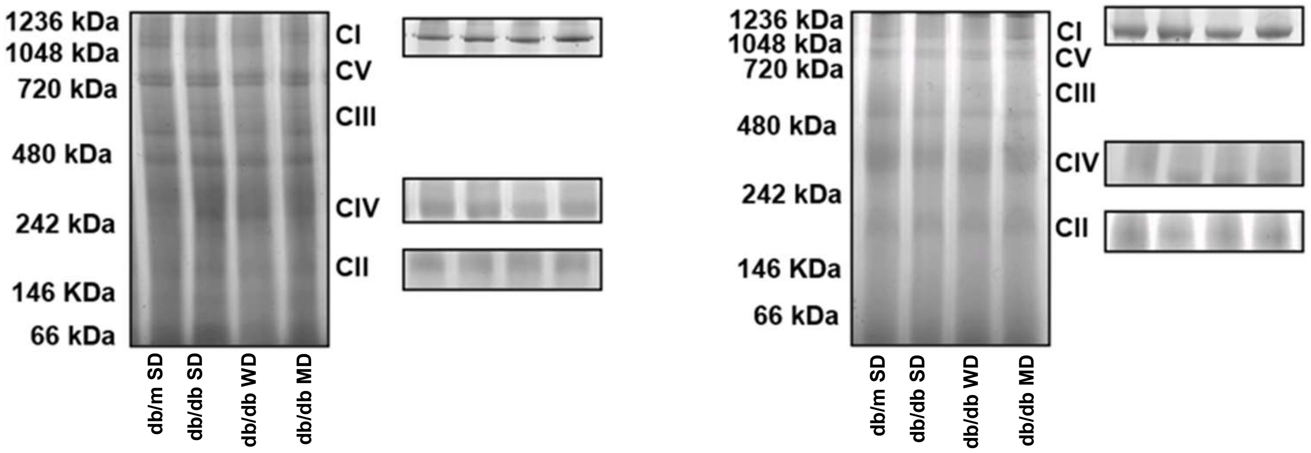

Supporting Information Figure S2. Representative images of BN-PAGE gels and histochemical staining of complex I (CI), complex IV (CIV), and complex II (CII) in-gel activity.

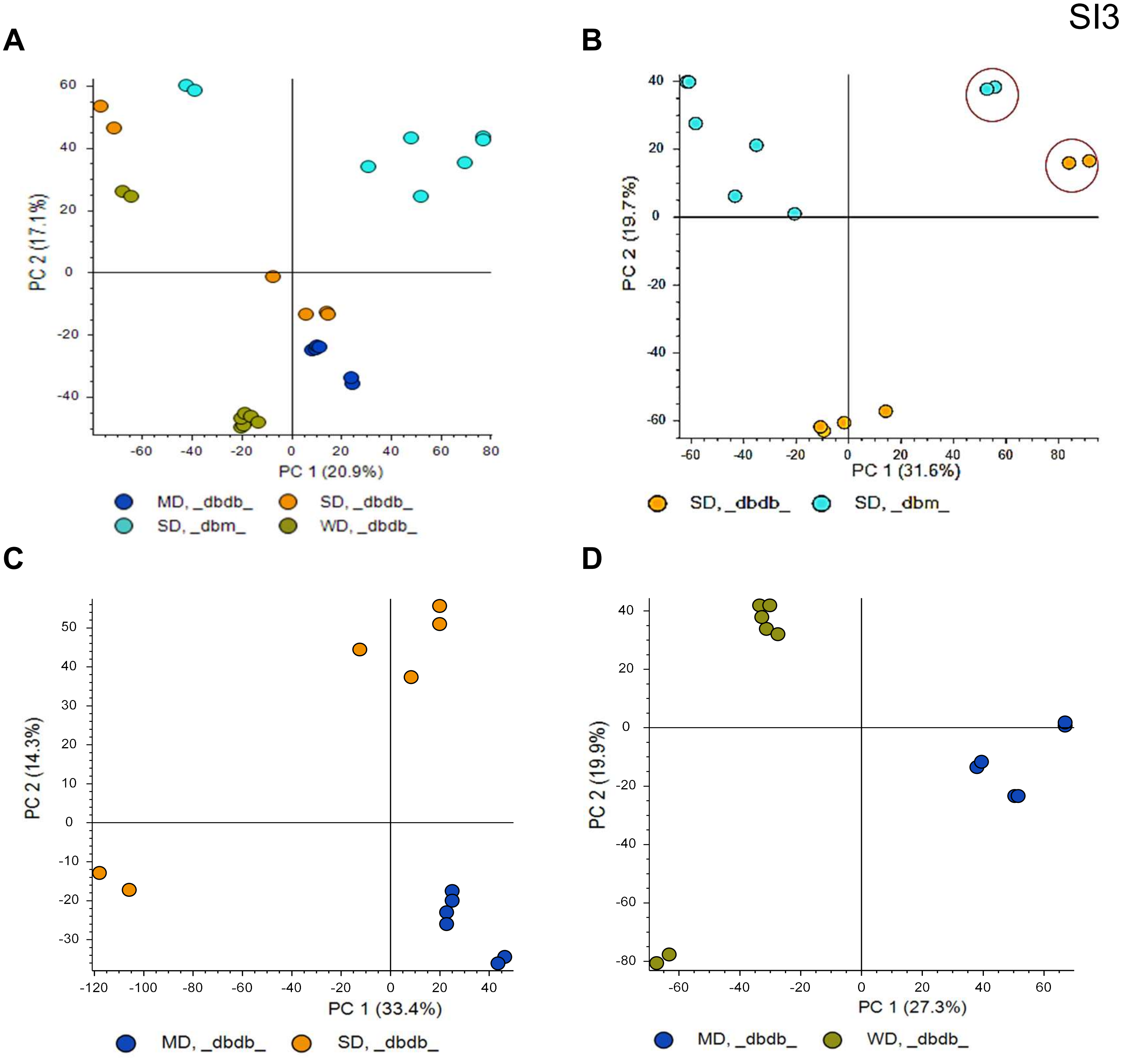

Supporting Information Figure S3. Principal component analysis (PCA) of the metabolomic data recorded for liver tissues from db/m and db/db mice experiencing a different diet. (A) PCA revealed a clear separation between control (db/m) and diabetic (db/db) mice along principal component 2 (PC2), except for two outliers in the WD-fed and SD-fed db/db mice groups. Among db/db mice, samples from MD-fed animals were distinctly separated from WD-fed counterparts along PC1, while samples from SD-fed mice showed no consistent clustering along PC1 or PC2. (B) In the SD-fed db/db mice vs. SD-fed db/m animal comparison, PCA showed significant changes in liver metabolomes, with clear PC2-based group discrimination, except for two db/db animal outliers. Comparisons of MD-fed vs. SD-fed db/db mice (C) and MD-fed vs. WD-fed db/db mice (D) also showed distinct separations along PC1 and PC2. Samples from SD-fed and WD-fed db/db mice were separated along PC2, with SD ones also showing PC1 separation.

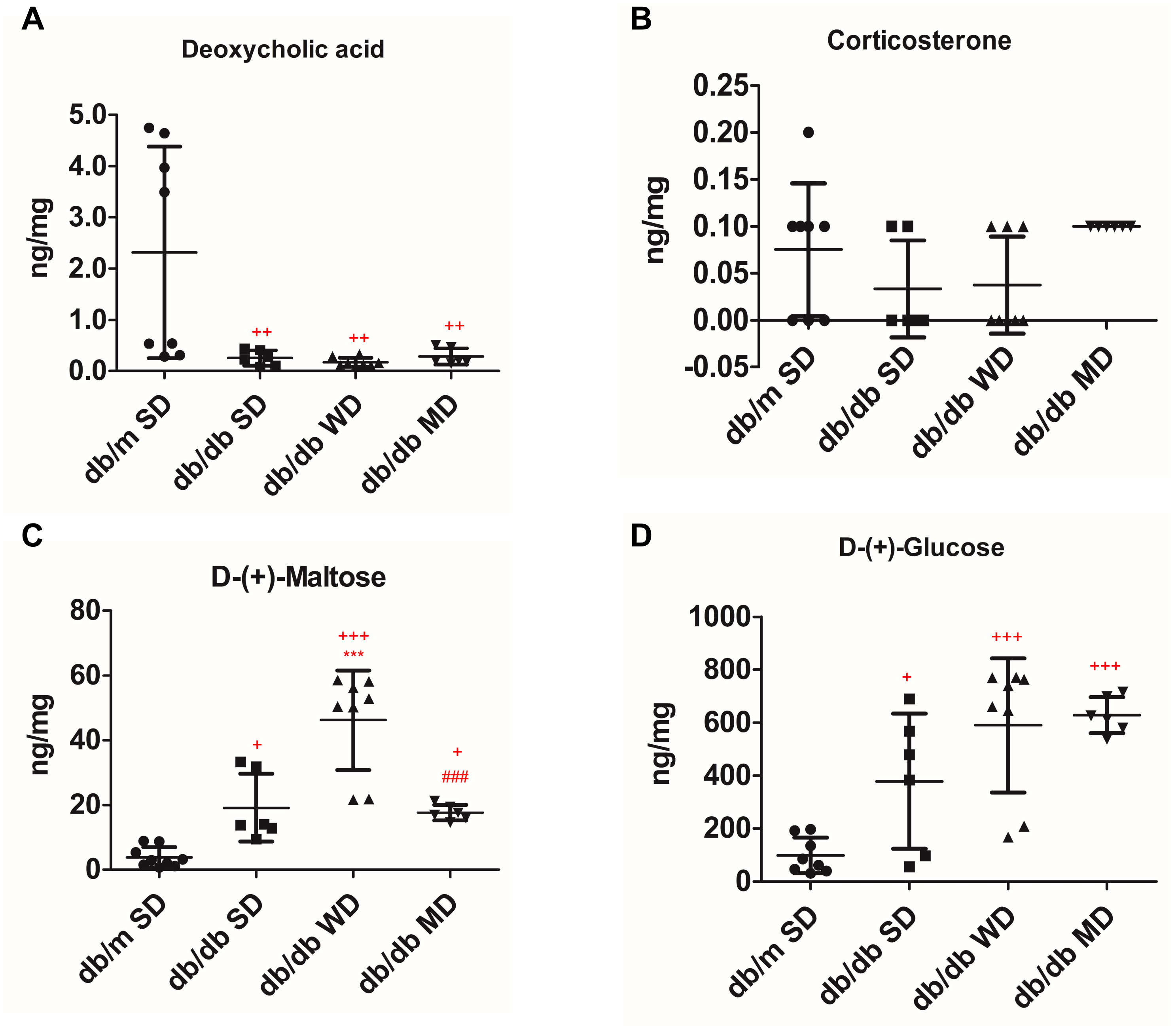

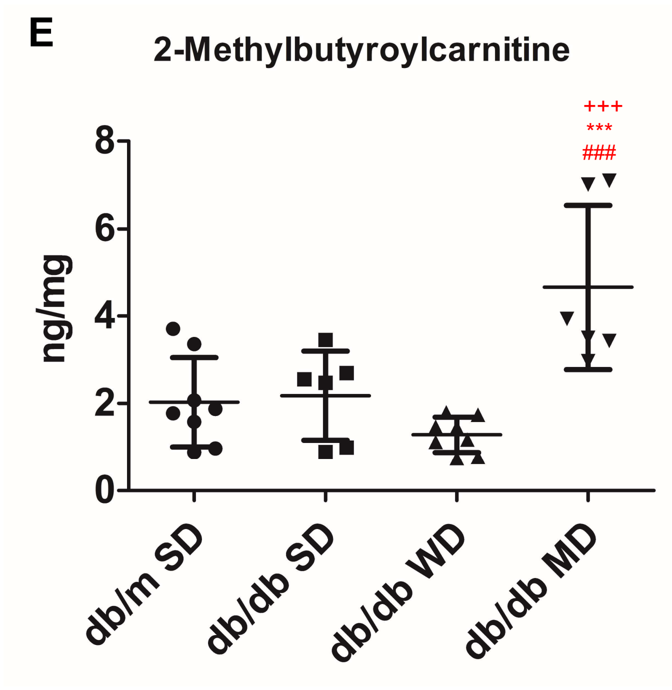

Supporting Information Figure S4. Effects of tested diets on selected liver metabolites. (A) deoxycholic acid; (B) corticosterone; (C), D-(+)-maltose; (D) D-(+)-glucose; (E) 2-methylbutyroylcarnitine. Shown are the mean values ± SD (ng/mg). n=6/8. One-way ANOVA, ++ p <0.01, +++ p <0.001 vs. SD-fed db/m mice; * p <0.05, ** p<0.01, *** p <0.001 vs. SD-fed db/db mice; ### p <0.001 vs. WD-fed db/db mice.
